# Evidence for an early innate immune response in the motor cortex of ALS

**DOI:** 10.1186/s12974-017-0896-4

**Published:** 2017-06-26

**Authors:** Javier H. Jara, Barış Genç, Macdonell J. Stanford, Peter Pytel, Raymond P. Roos, Sandra Weintraub, M. Marsel Mesulam, Eileen H. Bigio, Richard J. Miller, P. Hande Özdinler

**Affiliations:** 1 Department of Neurology and Clinical Neurological Sciences, Northwestern University Feinberg School of Medicine, 303 E. Chicago Ave. Ward 10-120, Chicago, IL 60611 USA; 2 Department of Pathology, University of Chicago Medical Center, Chicago, IL 60637 USA; 3 Department of Neurology, University of Chicago Medical Center, Chicago, IL 60637 USA; 4 Cognitive Neurology and Alzheimer’s Disease Center, Northwestern University, Chicago, IL 60611 USA; 5 Molecular Pharmacology and Biological Chemistry, Northwestern University Feinberg School of Medicine, Chicago, IL 60611 USA; 6 Robert H. Lurie Cancer Center, Northwestern University, Chicago, IL 60611 USA

**Keywords:** Upper motor neurons, Microglia, MCP1, CCR2

## Abstract

**Background:**

Recent evidence indicates the importance of innate immunity and neuroinflammation with microgliosis in amyotrophic lateral sclerosis (ALS) pathology. The MCP1 (monocyte chemoattractant protein-1) and CCR2 (CC chemokine receptor 2) signaling system has been strongly associated with the innate immune responses observed in ALS patients, but the motor cortex has not been studied in detail.

**Methods:**

After revealing the presence of MCP1 and CCR2 in the motor cortex of ALS patients, to elucidate, visualize, and define the timing, location and the extent of immune response in relation to upper motor neuron vulnerability and progressive degeneration in ALS, we developed MCP1-CCR2-hSOD1^G93A^ mice, an ALS reporter line, in which cells expressing MCP1 and CCR2 are genetically labeled by monomeric red fluorescent protein-1 and enhanced green fluorescent protein, respectively.

**Results:**

In the motor cortex of MCP1-CCR2-hSOD1^G93A^ mice, unlike in the spinal cord, there was an early increase in the numbers of MCP1+ cells, which displayed microglial morphology and selectively expressed microglia markers. Even though fewer CCR2+ cells were present throughout the motor cortex, they were mainly infiltrating monocytes. Interestingly, MCP1+ cells were found in close proximity to the apical dendrites and cell bodies of corticospinal motor neurons (CSMN), further implicating the importance of their cellular interaction to neuronal pathology. Similar findings were observed in the motor cortex of ALS patients, where MCP1+ microglia were especially in close proximity to the degenerating apical dendrites of Betz cells.

**Conclusions:**

Our findings reveal that the intricate cellular interplay between immune cells and upper motor neurons observed in the motor cortex of ALS mice is indeed recapitulated in ALS patients. We generated and characterized a novel model system, to study the cellular and molecular basis of this close cellular interaction and how that relates to motor neuron vulnerability and progressive degeneration in ALS.

**Electronic supplementary material:**

The online version of this article (doi:10.1186/s12974-017-0896-4) contains supplementary material, which is available to authorized users.

## Background

Amyotrophic lateral sclerosis (ALS) is a multisystem disorder in which both corticospinal motor neurons (CSMN; a.k.a. upper motor neurons or Betz cells in human) and spinal motor neurons (SMN) progressively degenerate. This leads to failure of the motor neuron circuitry and the neuromuscular system with loss of voluntary movement [1]. Even though ALS is manifested by progressive degeneration of motor neurons, the role and the impact of non-neuronal cells in the environment have been suggested as being important for both for the initiation and progression of the disease [2] and they are now considered as an integral part of disease pathology in ALS [3].

Increases in the innate immune response or microgliosis, as well as astrogliosis have been widely demonstrated in ALS patients, especially in the spinal cord [4–7]. Various animal models have been generated for investigating disease mechanisms of ALS, and the hSOD1^G93A^ mouse is one of the best-characterized models for ALS, in which both CSMN and SMN progressively degenerate and many disease pathologies, such as increased innate immune response, are recapitulated [8–10]. In this model, there is an increase of reactive astrocytes in close proximity to SMN in the spinal cord, during end stage [11, 12]. Although, astrocytes react in response to the dying SMN, evidence has suggested their involvement in promoting disease initiation and progression as well [13, 14]. More interestingly, the pivotal role of microglia activation as the main component of the innate immune response observed in ALS, in motor neuron degeneration as well as recruitment of monocytes and T cells to sites of injury, remains under debate [15]. It has been proposed that some of these immune effectors could be protective during disease onset, but ultimately a vicious cycle is generated towards the end stage, which promotes and facilitates toxic neuroinflammatory processes in the spinal cord [2, 16, 17].

Even though the spinal component of the motor neuron circuitry has been studied with much greater detail, there is developing interest in understanding the role of innate immunity and microgliosis in the motor cortex. Possible involvement of microgliosis in upper motor neuron pathology is suggested by earlier work demonstrating their presence in the motor cortex obtained from postmortem samples isolated from end-stage ALS patients [6] and imaging studies in ALS patients [18–21]. However, the timing and the extent of their involvement remained unknown, resulting in a debate as to whether or not they contribute to motor neuron degeneration or if they are a by-product of an ongoing neuronal death and cellular pathology. Investigation of ALS mouse models has hinted that an immune response may be initiated early as activated astrocytes and microglia were detected near the vulnerable CSMN [8, 22], but the basis of their cellular interaction or the details of the interplay between CSMN and the non-neuronal cells remained largely unknown. We hypothesize that the innate immune response could indeed play a role in CSMN vulnerability and progressive degeneration via accumulation and secretion of a select set of ultimately toxic inflammatory cytokines.

Monocyte chemoattractant protein-1 (MCP1) is an inducible chemokine expressed at very low levels in central nervous system (CNS) under normal conditions but expressed at high levels in the spinal cord within glia, macrophages, vasculature and anterior and dorsal horn motor neurons [23, 24]. MCP1 is also present at high levels in the CSF and plasma of ALS patients [24, 25]. Although, in ALS mouse models, MCP1 is present early in disease prior to microglial activation in the spinal cord [26], the role of MCP1-expressing microglia in recruitment of MCP1 receptor CCR2+ (CC chemokine receptor 2) monocytes to the spinal cord in hSOD1^G93A^ mice remains controversial [27, 28]. However, studies in ALS patients demonstrate dysregulation of circulating monocytes [29, 30], with decreased CCR2 levels in plasma possibly due to recruitment of “classical” (CD14+/CD16+/CCR2 high) monocytes into CNS [31, 32]. These studies clearly suggest the relevance of the MCP1-CCR2 system in the pathogenesis of ALS and the necessity of further studies to fully understand and characterize the role of the innate immune response, MCP1/CCR2 signaling in particular, with respect to pathogenesis of ALS.

In an effort to visualize MCP1-CCR2 interactions with motor neurons at different stages of the disease and to reveal the landscape of this innate immunity associated chemokine/receptor system throughout CNS and with respect to motor neuron vulnerability and progressive degeneration, we took advantage of a bitransgenic mouse model in which MCP1 and CCR2 are genetically labeled with monomeric red fluorescent protein-1 (mRFP) and enhanced green fluorescent protein (eGFP), respectively [33]. We crossed this bitransgenic mouse with the hSOD1^G93A^ mouse model of ALS to generate MCP1-CCR2-hSOD1^G93A^ mice, a triple transgenic ALS mouse line in which MCP1- and CCR2-expressing cells are fluorescently labeled in the hSOD1^G93A^ background. This novel disease reporter line enabled us to visualize and assess the timing and the extent of innate immune response occurring in the motor cortex and the spinal cord during disease initiation and progression. Our findings revealed that an innate immune response was an early event and continued to increase with disease progression. Analysis of postmortem tissue from end-stage ALS patients displayed presence of immune response especially in the motor cortex of both familial ALS (fALS) and sporadic ALS (sALS) patients, and cell-cell interaction primarily along the apical dendrites of diseased Betz cells.

## Methods

### Postmortem human brain samples

Postmortem human tissue collected according to protocols approved by an institutional review board was obtained from University of Chicago and Northwestern University. Clinical records were available for every subject. A neurologist examined all the patients and a neuropathologist with expertise in neurodegenerative disorders. Brains were fixed either in 10% neutral buffered formalin for 2 weeks or 4% paraformaldehyde (PFA) at 4 °C for 30 h, and sections were paraffin embedded. Areas of the primary motor cortex were retrieved, 4 μm thick serial sections were cut, mounted on a charged glass slide (Fisher Scientific), and used for immunocytochemical analyses. In this study, motor cortex isolated from normal control subjects with no neurologic disease and ALS patients were included. Detailed information about the normal controls and ALS cases can be found in Table 1.

**Table 1:** Information of postmortem motor cortex samples included in this study

Clinical diagnosis	Case	Age, years	Sex	Postmortem interval, h
Normal control	1	45	F	15
Normal control	2	60	M	19
Normal control	3	59	M	12
Normal control	4	64	F	6
Normal control	5	67	M	5
Normal control	6	75	F	17
Normal control	7	88	M	27
Normal control	8	100	F	14
Normal control	9	87	M	16
Sporadic ALS	1	49	F	15
Sporadic ALS	2	61	M	13
Sporadic ALS	3	61	M	13
Sporadic ALS	4	64	F	9
Sporadic ALS	5	66	F	12
Sporadic ALS	6	55	M	20
Sporadic ALS	7	68	F	17
Sporadic ALS	8	66	M	18
Sporadic ALS	9	62	F	7
Sporadic ALS	10	68	F	19
Familial ALS (SOD1 I113T)	1	79	F	24
Familial ALS (SOD1 E100G)	2	66	M	4
Familial ALS (SOD1 G93A)	3	64	M	18
Familial ALS (SOD1 I113T)	4	53	M	7
Familial ALS (SOD1 V148G)	5	50	F	4

Case numbers, age, sex and diagnosis of patients are included

### Mice

All procedures were approved by the Northwestern University Animal Care and Use Committee and conformed to the standards of the National Institutes of Health. hSOD1^G93A^ transgenic ALS mice, overexpressing the human SOD1^G93A^ mutation (B6SJL-Tg (SOD1*G93A)1Gur/J), were obtained from Jackson laboratories [10] and crossed with C57BL/6 mice for at least six generations to bring mice to the C57BL/6 background, and all mice were kept in this background. Genotypes of mice were determined by PCR, as described by the vendor. Reporter transgenic lines for MCP1 and CCR2 were obtained from Dr. Richard J. Miller. [33]. Bitransgenic mice containing CCR2-eGFP (CCR2 protein reporter) and mRFP (MCP1 transcriptional reporter) were crossed with the hSOD1^G93A^ mice to obtain MCP1-CCR2-hSOD1^G93A^ and MCP1-CCR2-WT (littermate controls) for at least six generations before used for experiments.

### CSMN retrograde transduction surgeries

All surgeries were performed as previously described [22]. Briefly, MCP1-CCR2-hSOD1^G93A^ (*N* = 6) and MCP1-CCR2-WT mice (*N* = 6) were deeply anesthetized with isoflurane and placed onto a stereotaxic platform equipped with a nanojector (Drummond Scientific, PA). A small laminectomy was performed at the cervical spinal cord, and adeno-associated virus encoding eGFP (AAV-eGFP; University of Pennsylvania) was injected into the corticospinal tract (CST) that lies within the dorsal funiculus (df) at 0.3 mm depth. Mice were injected at P30 with a volume of 621 nl containing 1.16 × 10^9^ transducing units of AAV encoding eGFP (AAV2-eGFP) and sacrificed at P60. AAV vector was generated by the University of Pennsylvania Vector Core facility as previously described [22].

### Tissue isolation and processing

Brain and spinal cord tissues were collected from MCP1-CCR2-hSOD1^G93A^ and MCP1-CCR2-WT mice at P30 (pre-symptomatic), P60 (early symptomatic), P90 (late symptomatic), and P120 (end stage). Mice were deeply anesthetized by ketamine (90 mg/kg) with xylazine (10 mg/kg) and transcardially perfused with 4% PFA in phosphate buffered saline (PBS). The brain and spinal cord were removed and were postfixed (4% PFA, overnight) and kept in PBS-sodium azide (0.01%) at 4 °C. Coronal (50 μm) serial sections were collected in 12-well plates using a vibratome (VT1000S, Leica Instruments).

### LPS treatment

MCP1-CCR2-hSOD1^G93A^ mice were injected intraperitoneally (I.P.) with 50 mg/kg body weight of lipopolysaccharide (LPS) diluted in saline at P60. Mice were sacrificed 6 h post treatment as previously described [34].

### Immunohistochemistry

Anti-CD11b (1:1000; Serotec), anti-GFAP (1:2000; Invitrogen), anti-GFP (1:1000; Abcam), anti-Iba1 (1:500; Wako), anti-Iba1 (1:500; Abcam), and anti-Ly6C (Abcam 1:1000), anti-NeuN (1:1000, Millipore), anti-RFP (1:500; MBL International), anti-MCP1 (1:100; Novus), anti-CD68 (1:200; Serotec), anti-CD45 (1:200, BD Biosciences), anti-Arg1 (1:100; Cell Signaling), and anti-iNOS (1:200; BD Biosciences) were used for immunohistochemical analysis as previously described [22]. Fluorescent secondary antibodies were purchased from Molecular Probes unless otherwise noted. Goat anti-chicken Alexa Fluor 488 (1:1000), goat anti-rabbit Alexa Fluor 488 (1:1000), donkey anti-rabbit Alexa Fluor 555, goat anti-mouse Alexa Fluor 647 (1:1000), and goat anti-rat Alexa Fluor 647 (1:1000) were used. A standard immunostaining protocol was performed for all antibodies unless otherwise specified. Briefly, free-floating sections were blocked with blocking solution (PBS, 0.05% BSA, 2% fetal bovine serum FBS, 1% Triton X-100, and 0.1% saponin) for 30 min at room temperature (RT) followed by overnight incubation at 4 °C with primary antibody/ies diluted in blocking solution. Primary antibody/ies were washed with PBS three times followed by 2 h incubation at RT with the appropriate secondary antibody/ies diluted in blocking solution. Secondary antibody/ies were washed with PBS three times, and sections were mounted on slides and coversliped with Fluoromount G (Electron Microscopy Sciences). For MCP1 immunostaining in combination with other antibodies, antigen retrieval with 0.01 M sodium citrate pH = 9 for 3 h in a water bath at 80 °C was performed prior to blocking and same procedure for primary and secondary antibody incubation was followed as abovementioned. For iNOS immunostaining in combination with other antibodies, antigen retrieval as described above and MOM kit was used according to the manufacturer’s instructions (Vector Laboratories). Briefly, sections were blocked with MOM blocking solution for 1 h, rinsed with PBS, incubated with MOM diluent for 10 min, and incubated with anti-iNOS diluted in MOM diluent for 30 min. Then MOM biotinylated antibody diluted in MOM blocking solution was applied, rinse with PBS, and incubated with Streptavidin-AlexaFluor647 for 10 min. After washes in blocking solution, the standard immunostaining protocol to detect other antibodies was used. For Ly6C/CD45/GFP and Arg1 immunostaining in combination with other antibodies, Fab fragments were used to change antibody species. Briefly, sections were blocked with blocking solution for 30 min at room temperature (RT) followed by overnight incubation at 4 °C with Rat (Rt) anti-Ly6C or Rabbit (Rb) anti-Arg1 diluted in blocking solution. Primary antibodies were washed with PBS three times followed by 2 h incubation at RT with goat anti-Rt or goat anti-Rb Fab fragments (Jackson ImmunoResearch Laboratories). After three PBS washes, sections were incubated with secondary anti-Gt AlexaFluor647 2 h incubation at RT. After washes in PBS, the standard immunostaining protocol to detect other antibodies was used.

### Immunohistochemistry in postmortem human samples

Slides were baked for 60 min at 60 °C, deparafinized with xylene for 5 min, rehydrated in ethanol (100, 95, 70, and 50%). For antigen retrieval, slides were immersed in 10 mM sodium citrate and subjected to high heat and pressure for 20 min. After cooling, slides were rinsed with PBS for 10 min and blocked with 0.5% bovine albumin serum, 0.1% Triton X-100, and 2% fetal bovine serum in PBS for 30 min, and incubated overnight at 4 °C with a mix of primary antibodies anti-Map2 (1:200, Millipore, Temecula, CA), anti-Iba1 (1:500; Wako), anti-GFAP (1:2000; Invitrogen), anti-MCP1 (1:100; Novus). After PBS rinses, slides were incubated with donkey anti-chicken Alexa Fluor 488 (1:1000), donkey anti-rabbit Alexa Fluor 555, and donkey anti-rat Alexa Fluor 647 (1:1000), conjugated secondary antibodies diluted in the blocking solution for 2 h at RT. Slides were rinsed in PBS and counterstained with DAPI. Autofluorescence was quenched with True Black (Biotum) according to manufacturer’s instructions and coversliped with Fluoromount G (Electron Microscopy Sciences).

### Imaging and quantification

For quantification purposes, images were taken using Nikon Eclipse TE2000-E fluorescence microscope with a ×20 objective. Confocal images were collected either using a Zeiss 510 Meta confocal microscope or Zeiss 880 confocal microscope (Zeiss). For quantitative analyses of MCP1+ cells in the brain, five comparable brain sections in the MCP1-CCR2-hSOD1^G93A^ and MCP1-CCR2-WT mice (*N* = 5) were chosen to comprise the majority of the motor cortex based on the mouse brain atlas including plate 21 (Bregma 1.18 mm), plate 25 (Bregma 0.74 mm), plate 30 (Bregma 0.14 mm), plate 35 (Bregma −0.46 mm), and plate 48 (Bregma −2.06 mm) [35]. The total number of MCP1+ and the percentage of CD11b+ cells was calculated in the motor cortex, somatosensory cortex, cingulate cortex, striatum, and hippocampus, at P30, P60, P90, and P120. Three sections were used for quantitative analyses of MCP1+ cells in the cervical and lumbar spinal cord of MCP1-CCR2-hSOD1^G93A^ and MCP1-CCR2-WT mice (*N* = 5). For quantitative analyses of Iba1+ cells in the human cortices, three random images per subject were taken using a 10× objective. For average number of Iba1+ cells, all Iba1+ cells that have a nucleus (DAPI+) were counted and averaged. For normal and activated microglia percentages, the numbers of normal and activated microglia were counted according to selection criteria previously described [36], and percentages of normal and activated were calculated individually and then averaged. All counts were performed blindly by same person, for consistency.

### Statistical analysis

All statistical analyses were performed using Prism software (version 5a; Graphpad Software Inc.). Statistical differences were determined by two-tailed unpaired *t* test previous D’Agostino & Pearson omnibus normality test or by Kruskal-Wallis test with Dunn’s multiple comparison test. Statistically significant differences were considered at least *p* < 0.05, and values were expressed as the independent mean ± standard error of the mean (SEM).

## Results

### Evidence of microgliosis in the vicinity of Betz cells in ALS

**Fig. 1:**
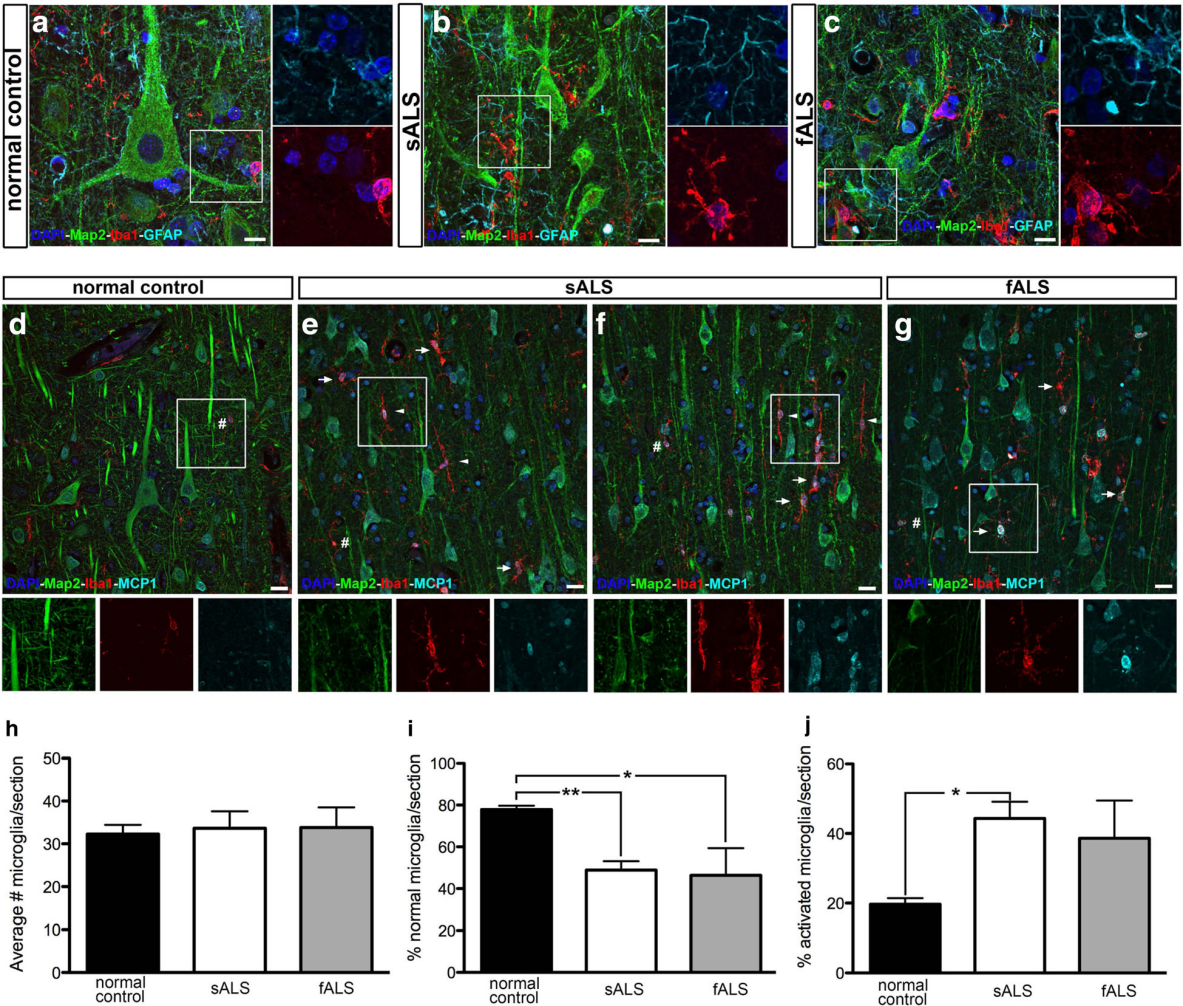
Activated microglia express MCP1 in the motor cortex of ALS patients. Representative images of layer V of the motor cortex isolated from postmortem normal controls and ALS patients. **a** Normal controls have large Betz cells (Map2+) located in layer V of the motor cortex, with no evidence of gliosis. *Inset* is enlarged to the right. **b**, **c** sALS and fALS cases have smaller Betz cells (Map2+) and microgliosis (Iba1+), and astrogliosis (GFAP+) is observed. *Insets* are enlarged to the right. **d** Normal controls have low levels of MCP1 expression. *Inset* is enlarged at the bottom. **e**–**g** sALS and fALS cases have high levels of MCP1, and MCP1+ cells co-localize with activated microglia (Iba1+). *Insets* are enlarged at the bottom. **h** *Graph* represents average number or microglia per section ± SEM in normal controls (*black column*), sALS (*white column*), and fALS (*grey column*). **i** *Graph* represents percentage of normal microglia per section ± SEM in normal controls (*black column*), sALS (*white column*), and fALS (*grey column*). **j** *Graph* represents percentage of activated microglia per section ± SEM in normal controls (*black column*), sALS (*white column*), and fALS (*grey column*). *p < 0.05, **p < 0.01. *Number sign* marks normal microglia; *arrows* point to activated microglia; *arrowheads* point to rod-like microglia. Abbreviations: *sALS* sporadic ALS, *fALS* familial ALS. *Scale bar* = 10 μm (**a**–**c**), *scale bar* = 20 μm (**d**–**g**)

We first investigated the occurrence of microgliosis or astrogliosis in the vicinity of Betz cells located at layer V of the motor cortex of patients with sALS and fALS with SOD1 mutations (Fig. 1). In normal controls, Betz cells had large cell bodies with healthy apical dendrites (Fig. 1a), and microglia (Iba1+) and astrocytes (GFAP+) were present in their vicinity but did not display signs of activation. In both sALS and fALS subjects, Betz cells appeared smaller in size, and gliosis was observed throughout the motor cortex (Fig. 1b, c), as previously reported [4, 37], although the overall numbers of microglia were not increased (Iba1+; average number of microglia per section, normal control 32 ± 6, *N* = 9; sALS 34 ± 13, *N* = 10; fALS 34 ± 10, *N* = 5; Fig. 1h).

To distinguish between quiescent and activated microglia and to investigate a potential shift in their activation state, previously described criteria was used [36]. “Normal” microglia were defined by a typically ramified appearance with short and fine processes, whereas “activated” microglia were defined by short and protruding processes. Normal controls contained mostly normal microglia (Fig. 1a, d, i). Conversely, in ALS cases, microglia displaying an activated phenotype were increasingly present in close proximity to and at times surrounding, Betz cells and their apical dendrites (Fig. 1b, c, e–g arrows). The percentage of normal microglia was significantly reduced in both sALS and fALS cases (normal controls 78 ± 2%, *N* = 9; sALS 49 ± 4%, *N* = 10, *p* < 0.01; fALS 46 ± 13%, *N* = 5, *p* < 0.05; Fig. 1i). This decrease in the percentage of normal microglia was accompanied by an increase in the percentage of activated microglia in ALS cases being significantly higher in sALS (normal control 20 ± 2%, *N* = 9; sALS 44 ± 5%, *N* = 10, *p* < 0.01; fALS 39 ± 11%, *N* = 5; Fig. 1j). In addition, abnormal rod-like microglia often fused with each other and characterized by remarkably elongated nuclei were also observed only in ALS cases (normal controls 2 ± 0.5%, *N* = 9; sALS 6 ± 2%, *N* = 10; fALS 1 ± 0.4%, *N* = 5; Fig. 1e–g, arrowheads).

Since previous studies have suggested the involvement of MCP1 in ALS pathology [23, 24], we next investigated whether neurons and/or microglial cells in the motor cortex express MCP1. This analysis revealed low levels of MCP1 expression in both mature neurons (Map2+) and microglial cells (Iba1+) in normal controls (Fig. 1d). In contrast, high levels of MCP1 were detected in both patients with sALS and fALS, and MCP1 expression was present in neurons and all microglia being the most significant contributor to MCP1 expression in ALS motor cortices. These observations, displaying the occurrence of MCP1 in activated immune cells in the motor cortex of both sALS and fALS patients, indicate a potential involvement of MCP1/CCR2 axis in the motor cortex pathology observed in ALS.

### Generation of MCP1-CCR2-hSOD1^G93A^ triple transgenic mice

**Fig. 2:**
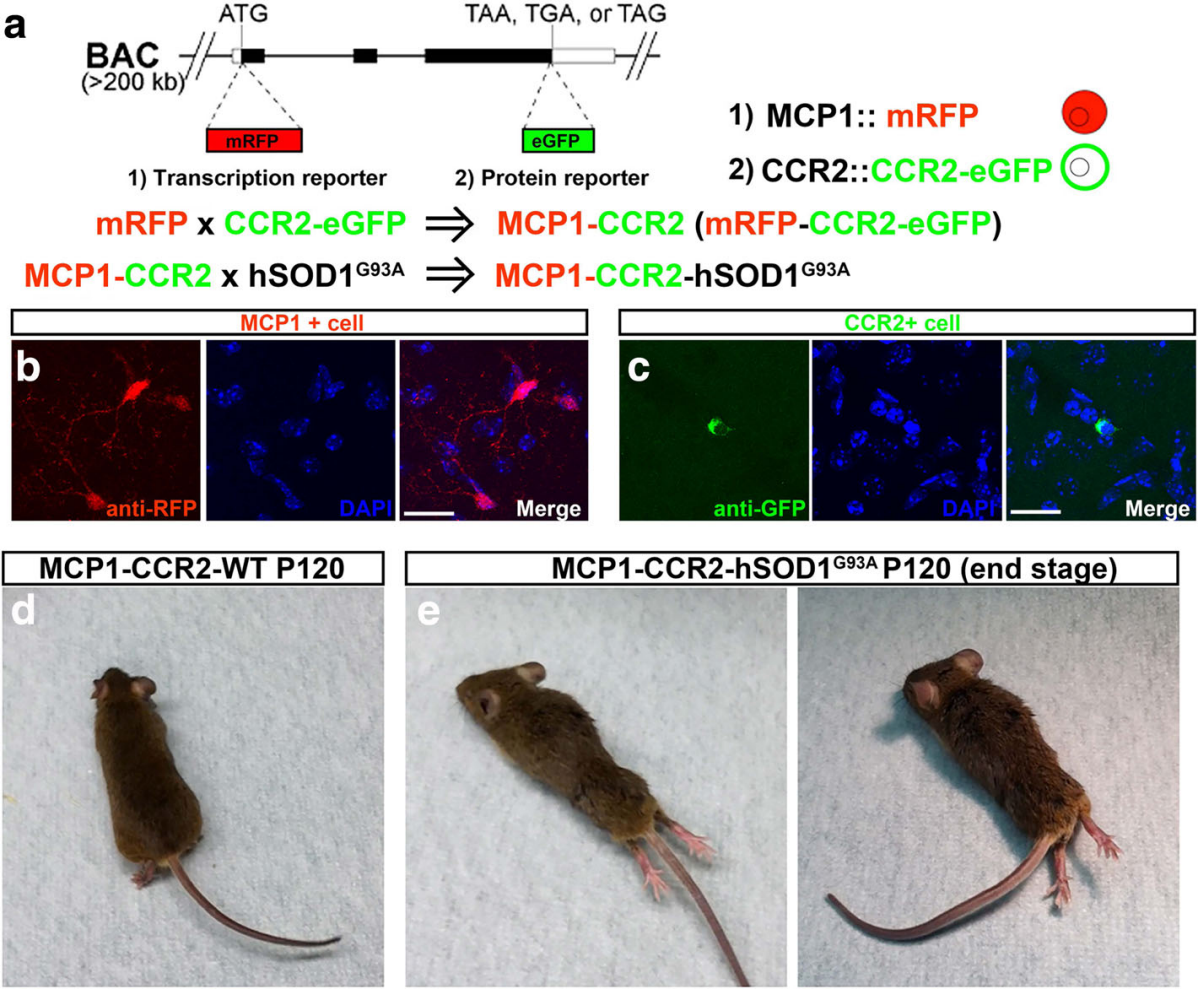
Generation of MCP1-CCR2-hSOD1^G93A^ triple transgenic mice. **a** BAC clone was modified by mRFP gene insertion in place of the MCP1 start codon (transcription reporter) and eGFP insertion in place of the CCR2 stop codon (protein reporter). MCP1-CCR2-hSOD1^G93A^ triple transgenic mice were generated upon multiple crosses between the MCP1-CCR2 mice and hSOD1^G93A^ mice. **b**, **c** MCP1+ and CCR2+ cells express eGFP and mRFP and can be visualized in vivo. **d** MCP1-CCR2-WT mice retain their health, while **e** MCP1-CCR2-hSOD1^G93A^ mice recapitulate ALS disease model as reported in the hSOD1^G93A^ mice. *Scale bar* = 20 μm

In an effort to investigate the role of MCP1 in the innate immune responses observed in ALS pathology, we generated MCP1-CCR2-hSOD1^G93A^ triple transgenic mice by multiple crosses between the MCP1-CCR2 chemokine-receptor bitransgenic mice [33] and hSOD1^G93A^ mice (Fig. 2a) so that in this triple transgenic mice, MCP1 and CCR2 expressing cells are fluorescently labeled in the hSOD1^G93A^ background. Littermates that do not express the hSOD1^G93A^ mutation (MCP1-CCR2-WT) were used as controls. MCP1-CCR2 transgenic mice are a reporter line in which the mRFP gene has been inserted in place of the start codon of MCP1 (transcription reporter, Fig. 2a), and eGFP has been inserted in place of the CCR2 stop codon (protein reporter, Fig. 2a) [33]. Since the MCP1-CCR2 bitrangenic mouse is a protein reporter for CCR2, eGFP expression is confirmatory for the presence of the fusion protein CCR2-eGFP [33], and MCP1 production/expression in mRFP+ cells was confirmed using anti-MCP1 immunohistochemistry (Additional file 1: Figure S1). The presence of MCP1+ and CCR2+ cells in the MCP1-CCR2 bitrangenic mice was confirmed with anti-RFP and anti-eGFP immunohistochemistry (Fig. 2b, c). Phenotypic assessment revealed that MCP1+ cells normally exhibited a ramified cellular morphology, and CCR2+ cells were round shape without ramifications. MCP1-CCR2-hSOD1^G93A^ triple transgenic mice recapitulated the well-defined disease pathology that have been previously observed and extensively characterized in the hSOD1^G93A^ mice [10]. The expression of fluorescently labeled MCP1 and CCR2 did not change disease progression in hSOD1^G93A^ mice; the timing and the progression rate of ALS were retained. The mice were pre-symptomatic at P30, began to show symptoms at P60, were fully symptomatic at P90, and reached disease end stage at P120. MCP1-CCR2-hSOD1^G93A^ mice continued to develop ALS and severe hind limb paralysis was most prominent during end stage, as previously reported in hSOD1^G93A^ mice (Fig. 2d, e). The presence of fluorescently labeled MCP1+ and CCR2+ cells in one of the most well-studied ALS model thus lay a strong foundation for the detailed cellular analysis of the innate immune response with respect to disease initiation and progression.

### Visualization of MCP1+ and CCR2+ cells in the brain and spinal cord

**Fig. 3:**
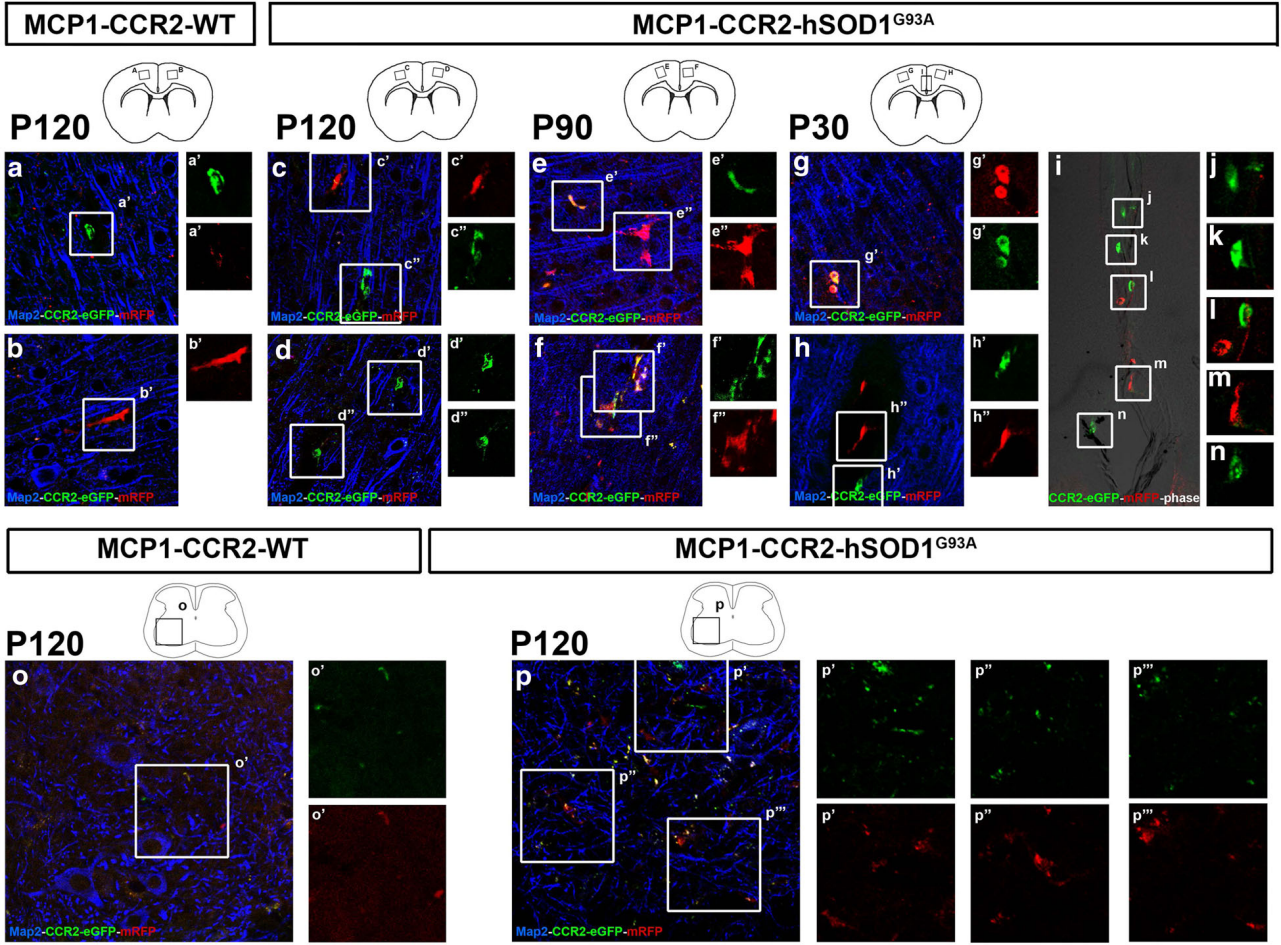
MCP1+ and CCR2+ cells are visualized in motor cortex and spinal cord of MCP1-CCR2-hSOD1^G93A^ mice. **a**, **b** Representative images of MCP1+ and CCR2+ cells with no immunohistochemistry enhancement for mRFP or eGFP. Very few cells are present in the motor cortex of MCP1-CCR2-WT mice. Insets enlarged to the right (*a’*, *b’*). **c**–**h** Representative images of MCP1+ and CCR2+ cells with no immunohistochemistry enhancement for mRFP or eGFP. Number of cells are present vary at different stages of disease initiation and progression throughout the motor cortex in MCP1-CCR2-hSOD1^G93A^ mice. *Insets* enlarged to the right (*c’*–*h”*). **i** Representative images of MCP1+ and CCR2+ cells with no immunohistochemistry enhancement for mRFP or eGFP. Cells are noticeable around the meninges surrounding the cingulate cortex in MCP1-CCR2-hSOD1^G93A^ mice. Insets enlarged to the right (**j**–**n**). **o** Representative images of MCP1+ and CCR2+ cells with no immunohistochemistry enhancement for mRFP or eGFP. Few cells are present in the ventral horn of the MCP1-CCR2-WT mice spinal cord. *Inset* enlarged to the right (*o’*). **p** Representative images of MCP1+ and CCR2+ cells with no immunohistochemistry enhancement for mRFP or eGFP. Numerous cells are present in MCP1-CCR2-hSOD1^G93A^ mice at the end stage. *Insets* enlarged to the right (*p’*–*p”’*). MCP1+ and CCR2+ cells do not co-localize with neuronal marker (Map2) in the motor cortex or spinal cord

We next evaluated whether MCP1+ and CCR2+ cells were visualized during different stages of disease progression in the brain and in the spinal cord of MCP1-CCR2-hSOD1^G93A^ mice. Initial observations in the motor cortex of MCP1-CCR2-WT mice suggested fewer MCP1+ or CCR2+ cells at all ages investigated (Fig. 3a, b displaying P120 as a representative example) in comparison to the presence of MCP1+ and CCR2+ cells in the motor cortex during disease progression (Fig. 3c–i). Using Map2 as a tool to label the neuronal component of the brain parenchyma, we detected MCP1+ and CCR2+ cells in large blood vessels and juxtaposed to the capillaries. Interestingly, they were abundantly present along the pia in the midline, suggesting a potential migration into the motor cortex in the MCP1-CCR2-hSOD1^G93A^ mice as early as P30 (Fig. 3i–n).

Degeneration of SMN accompanied by an immune response is a well-established phenomenon in hSOD1^G93A^ mice [15, 38, 39], and therefore we investigated the lumbar spinal cord of MCP1-CCR2-WT and MCP1-CCR2-hSOD1^G93A^ mice during disease progression. MCP1+ and CCR2+ cells were detected at all ages studied (Fig. 3o, p displaying P120 as a representative example), and MCP1+ and CCR2+ cells became very prominent during end stage (P120), which is in line with previous reports, revealing astrogliosis and microgliosis especially during end-stage disease [2].

**Fig. 4:**
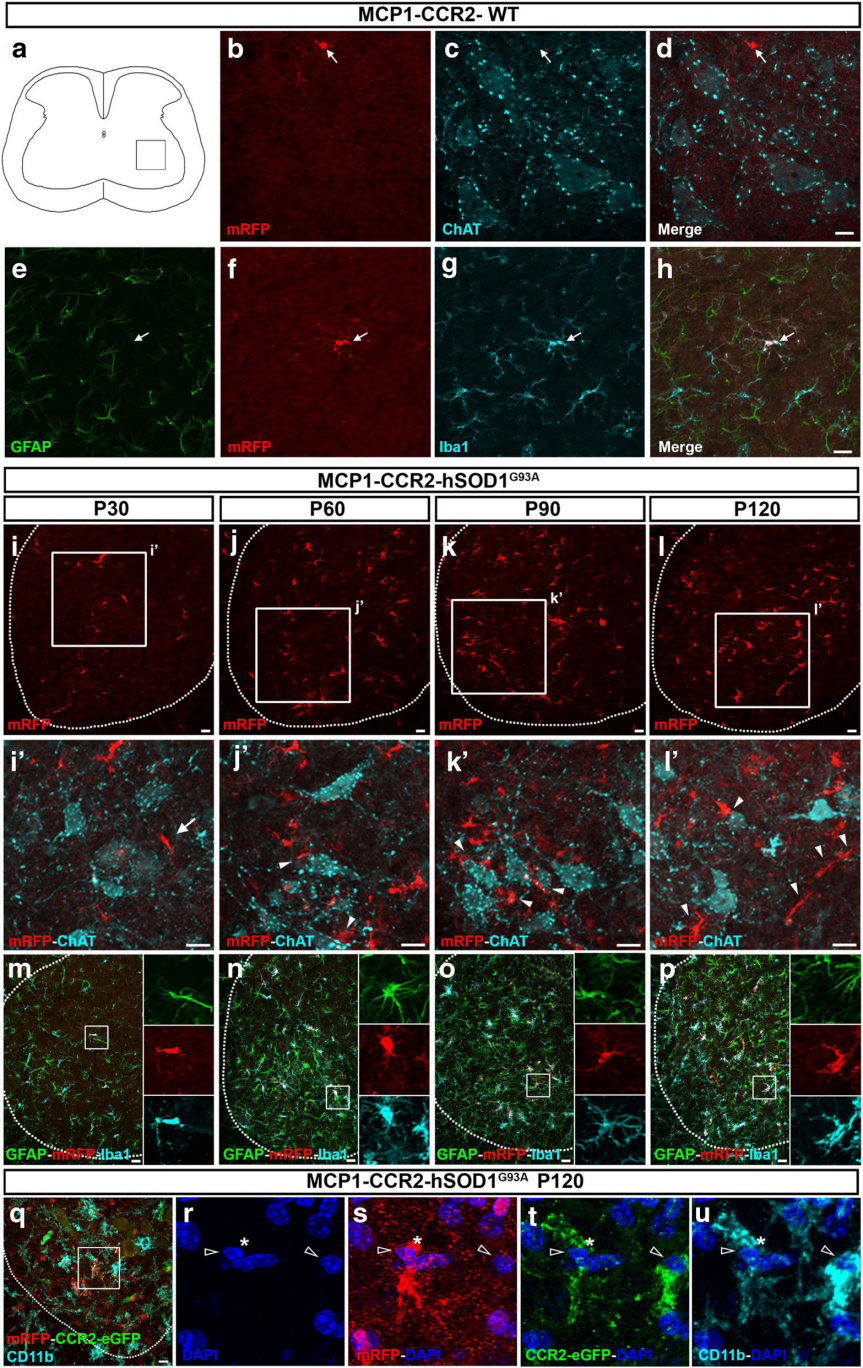
MCP1+ and CCR2+ cells are present during disease progress in MCP1-CCR2-hSOD1^G93A^ mice spinal cord. **a** Schematic drawing of lumbar spinal cord showing the area of the ventral horn used to obtain representative images. **b**–**d** Representative images of MCP1+ cells (*arrow*) in the ventral horn of the MCP1-CCR2-WT mice at P60 reveal that MCP1+ cells neither co-localize with SMN (ChAT+ neurons) nor they are in direct contact with SMN. **e**–**h** Representative images of MCP1+ cells (arrow) reveal sporadic presence of MCP1+ cells in MCP1-CCR2-WT at P60 that co-localize with Iba1+ microglia but not with GFAP+ astrocytes. **i**–**l** Representative images of the MCP1+ cells in the ventral horn of the MCP1-CCR2-hSOD1^G93A^ mice reveal their increase during disease progression. *Insets* are enlarged at the bottom (*i’*–*l’*). MCP1+ cells are in close proximity to SMN during pre-symptomatic stage (*arrows*) and closely interact with diseased SMN, especially at the end stage of the disease (*arrowheads*). **m**–**p** Representative images of the ventral horn of the MCP1-CCR2-hSOD1^G93A^ mice reveal the progressive microgliosis (Iba1+) and astrogliosis (GFAP+) and MCP1+ cells co-localized with microglia marker but not with astrocyte marker. *Insets* are enlarged to the right. **q**–**u** Representative images of the MCP1+ and CCR2+ cells in the ventral horn of the end-stage MCP1-CCR2-hSOD1^G93A^ mice show numerous MCP1+ cells (*asterisk*) that co-localize with monocyte/macrophage lineage marker (CD11b+). CCR2+ cells (*empty arrowheads*) co-localize with CD11b and MCP1+-CCR2+ cell-cell interactions are observed (**r**–**u**). *Dashed line* delineates the grey matter in the ventral horn. *Scale bar* = 20 μm

Even though MCP1+ and CCR2+ cells were detected in both MCP1-CCR2-WT and MCP1-CCR2-hSOD1^G93A^ mice without immunohistochemical enhancement (Fig. 3), we performed anti-RFP and anti-eGFP immunohistochemistry to increase the sensitivity and stability of fluorescence to obtain accurate cellular quantifications and to improve phenotypic assessment. First, we investigated the cellular identity, cellular morphology, and their relative location with respect to motor neurons of MCP1+ cells at different stages of the disease in the lumbar spinal cord (Fig. 4). In the MCP1-CCR2-WT mice, SMN were healthy and surrounded by scattered MCP1+ cells present in the spinal grey matter, although no evidence of cell-cell interactions was present (Fig. 4a–d, displaying P60 as a representative example). MCP1+ cells were small in size and displayed normal morphology of inactivated microglia, which was confirmed by co-localization with microglia maker Iba1 but not astrocyte marker GFAP (Fig. 4e–h). Both MCP1+ and Iba1+ cells confirmed lack of microgliosis in the MCP1-CCR2-WT mice. Conversely, MCP1+ cells were present at high numbers in the spinal cord of MCP1-CCR2-hSOD1^G93A^ mice during disease progression (Fig. 4i–l). Detailed cellular analysis in the ventral horn revealed cellular characteristics of activated microglia, with short and thick processes from the pre-symptomatic stage (Fig. 4i) with MCP1+ cells in the close vicinity of SMN (Fig. 4i’). Interaction of MCP1+ cells with degenerating SMN became more evident after symptomatic stage in the MCP1-CCR2-hSOD1^G93A^ mice (Fig. 4j’–l’ , arrowheads). Microgliosis and astrogliosis were also clear as disease progressed in the MCP1-CCR2-hSOD1^G93A^ mice as previously documented and MCP1+ cells co-localized with Iba1 (Fig. 4m–p). Microglial and microglia/monocyte lineage identity of MCP1+ cells at P60 in the spinal cord was also confirmed by quantification of Iba1 and CD11b expression, respectively. The majority of the MCP1+ cells were microglia (CD11b+/MCP1+; MCP1-CCR2-WT 90 ± 10%, *N* = 12, MCP1-CCR2-hSOD1^G93A^ 97 ± 1%, *N* = 113; and Iba1+/MCP1+; MCP1-CCR2-hSOD1-WT 89 ± 11%, *N* = 10; MCP1-CCR2-hSOD1^G93A^ 98 ± 2%, *N* = 121). Neither MCP1+ nor CCR2+ cells were astrocytes (GFAP+/MCP1+; MCP1-CCR2-hSOD1-WT 0%, *N* = 12; MCP1-CCR2-hSOD1^G93A^ 0%, *N* = 121), but interestingly, they were frequently found either in close proximity to or in a cell-cell contact with an astrocyte (Fig. 4m–p), suggesting an active cellular interaction, which warrants further detailed analysis. Since presence of MCP1+ and CCR2+ cells was prominent at the end stage, we also evaluated their cellular interactions (Fig. 4q–u). As previously described MCP1+ cells co-localized with monocyte lineage marker (CD11b+) (Fig. 4 s, u, asterisk). Cell-cell interactions between MCP1+ (asterisk) and CCR2+ (empty arrowheads) cells were also frequently observed, and CCR2+ cells co-localized with monocyte/macrophage lineage marker CD11b (Fig. 4t, u).

### Number of MCP1+ cells increases in the ventral horn of the spinal cord in the MCP1-CCR2-hSOD1^G93A^ mice

**Fig. 5:**
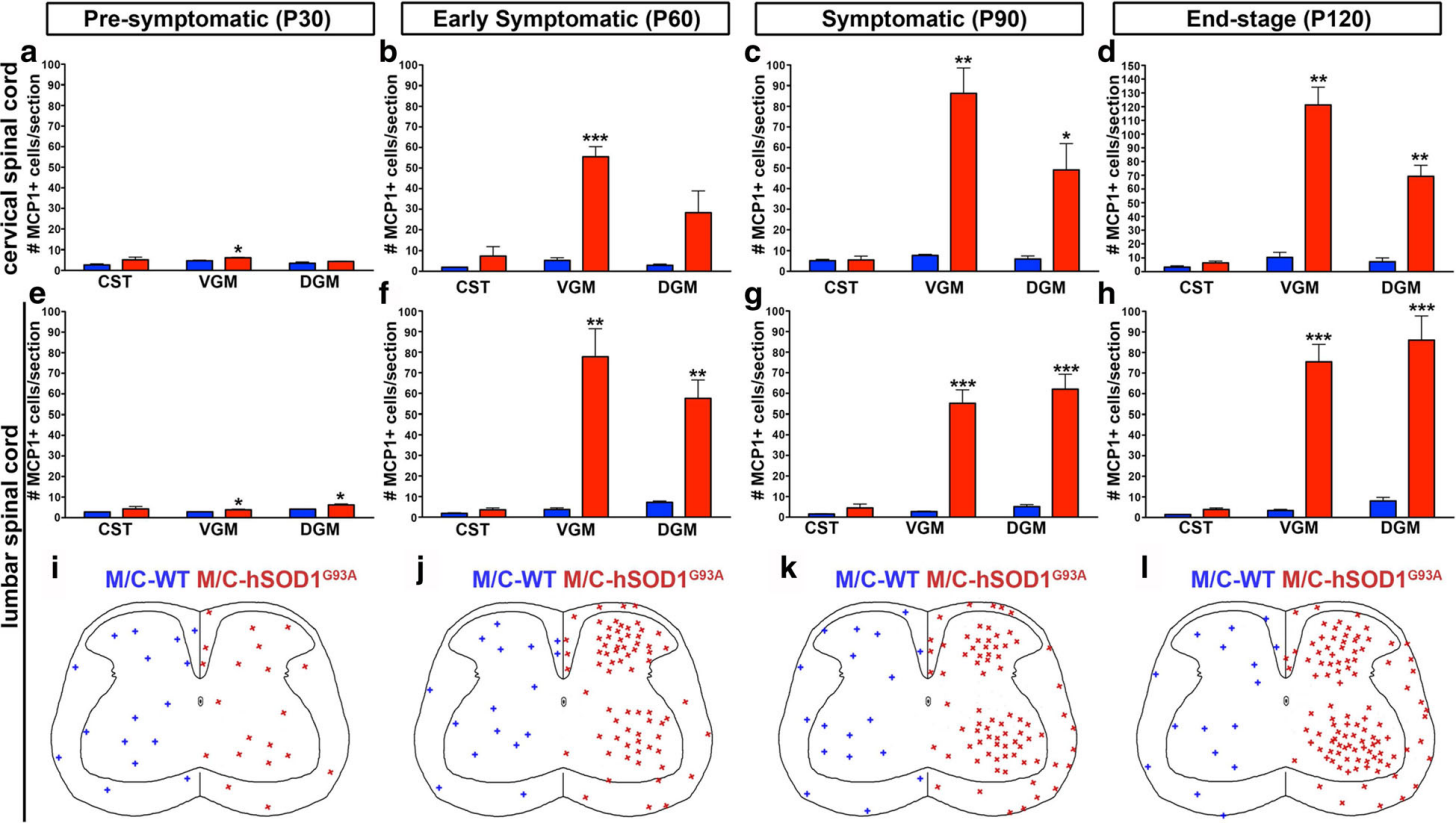
Number of MCP1+ cells increases during disease progression in the spinal cord of MCP1-CCR2-hSOD1^G93A^ mice. **a**–**h** *Graphs* represent average number of MCP1+ cells numbers per section ± SEM in MCP1-CCR2-WT mice (*blue columns*) and MCP1-CCR2-hSOD1^G93A^ triple transgenic mice (*red column*) in the cervical (**a**–**d**) and lumbar spinal cord (**e**–**h**) at the pre-symptomatic (P30), early symptomatic (P60), symptomatic (P90), and end stage (P120). **i**–**l** Representative drawings showing the relative location of MCP1+ cells in the three regions of the lumbar spinal cord studied. Abbreviations: *CST* corticospinal tract, *VGM* ventral grey matter, *DGM* dorsal grey matter. *p < 0.05, **p < 0.01, ***p < 0.01; MCP1-CCR2-WT mice vs. MCP1-CCR2-hSOD1^G93A^ triple transgenic mice

Since MCP1+ cells dramatically increased in the lumbar spinal cord of the MCP1-CCR2-hSOD1^G93A^ mice, we sought to investigate the timing of such increment of MCP1+ cells. For this purpose, we quantified the number of MCP1+ cells in the lumbar as well as the cervical spinal cord during disease initiation and progression. MCP1+ cells were quantified within the corticospinal tract (CST) region, ventral grey matter (VGM), and dorsal grey matter (DGM) in order to account for differences in three distinct regions of the spinal cord (Fig. 5) of MCP1-CCR2-hSOD1^G93A^ and MCP1-CCR2-WT mice (healthy control littermates).

The number of MCP1+ cells in the CST of MCP1-CCR2-WT mice was very low in both the cervical (P30: 3 ± 0.5, Fig. 5a; P60: 2 ± 0.1, Fig. 5b; P90: 5 ± 0.6, Fig. 5c, and P120: 3 ± 1, Fig. 5d) and lumbar spinal cord (P30: 3 ± 0.1, Fig. 5e, i; P60: 2 ± 0.3, Fig. 5 f, j; P90: 2 ± 0.1, Fig. 5g, k; and P120: 1 ± 0.02, Fig. 5h, l). Moreover, the numbers of MCP1+ cells at the site of the CST were comparable in both the cervical (P30: 5 ± 1, Fig. 5a; P60: 7 ± 4, Fig. 5b; P90: 5 ± 2, Fig. 5c, and P120: 6 ± 1, Fig. 5d) and lumbar spinal cord (P30: 4 ± 1, Fig. 5e, i; P60: 4 ± 1, Fig. 5 f, g; P90: 4 ± 2, Fig. 5g, k; and P120: 4 ± 1, Fig. 5h, l) in the MCP1-CCR2-hSOD1^G93A^ mice, and no statistical differences were detected.

The number of MCP1+ cells were also low in the VGM of MCP1-CCR2-WT in both in the cervical (P30: 5 ± 0.3, Fig. 5a; P60: 5 ± 1, Fig. 5b; P90: 8 ± 1, Fig. 5c, and P120: 10 ± 4, Fig. 5d) and lumbar spinal cord (P30: 3 ± 0.1, Fig. 5e, i; P60: 4 ± 1, Fig. 5f, j; P90: 3 ± 0.2, Fig. 5g, k; and P120: 3 ± 1, Fig. 5h, l). However, MCP1+ cell numbers in the VGM were increased in the MCP1-CCR2-hSOD1^G93A^ mice when compared to MCP1-CCR2-WT mice both in the cervical (P30: 6 ± 0.2, *p* = 0.0440, Fig. 5a; P60: 55 ± 5, *p* = 0.0006, Fig. 5b; P90: 86 ± 12, *p* = 0.0031, Fig. 5c; and P120: 121 ± 13, *p* = 0.0012, Fig. 5d) and lumbar spinal cord (P30: 4 ± 0.3 *p* = 0.0440, Fig. 5e, i; P60: 78 ± 14, *p* = 0.0013, Fig. 5f, j; P90: 55 ± 7, *p* = 0.0002, Fig. 5g, k; and P120: 75 ± 8, *p* = 0.0002, Fig. 5h, l).

Similarly, number of MCP1+ cells were also elevated in the DGM of the MCP1-CCR2-hSOD1^G93A^ mice both in the cervical (P30: MCP1-CCR2-WT: 4 ± 1 vs. MCP1-CCR2-hSOD1^G93A^: 4 ± 0.2, Fig. 5a; P60: MCP1-CCR2-WT: 3 ± 1 vs. MCP1-CCR2-hSOD1^G93A^: 28 ± 11, Fig. 5b; P90: MCP1-CCR2-WT: 6 ± 2 vs. MCP1-CCR2-hSOD1^G93A^: 49 ± 13, Fig. 5c, *p* = 0.0287; and P120: MCP1-CCR2-WT: 7 ± 3, MCP1-CCR2-hSOD1^G93A^: 69 ± 8, *p* = 0.0019, Fig. 5d) and lumbar spinal cord (P30: MCP1-CCR2-WT: 4 ± 0.1 vs. MCP1-CCR2-hSOD1^G93A^: 6 ± 0.5, *p* = 0.0186, Fig. 5e, i; P60: MCP1-CCR2-WT: 7 ± 1 vs. MCP1-CCR2-hSOD1^G93A^: 58 ± 9, *p* = 0.0011, Fig. 5f, j; P90: MCP1-CCR2-WT: 5 ± 1 vs. MCP1-CCR2-hSOD1^G93A^: 62 ± 7, *p* = 0.0002, Fig. 5g, k; P120: MCP1-CCR2-WT: 8 ± 2 vs. MCP1-CCR2-hSOD1^G93A^: 86 ± 12, *p* = 0.0006, Fig. 5h, l). These results demonstrate that MCP1+ cell numbers are comparable between MCP1-CCR2-hSOD1^G93A^ and MCP1-CCR2-WT mice at the site of CST throughout the disease but are significantly increased to similar extents mainly in the ventral horn, where SMN reside. The difference becomes much more evident in both the cervical and lumbar spinal cord, by P60.

### Number of MCP1+ cells increases early in the motor cortex in the MCP1-CCR2-hSOD1^G93A^ mice

**Fig. 6:**
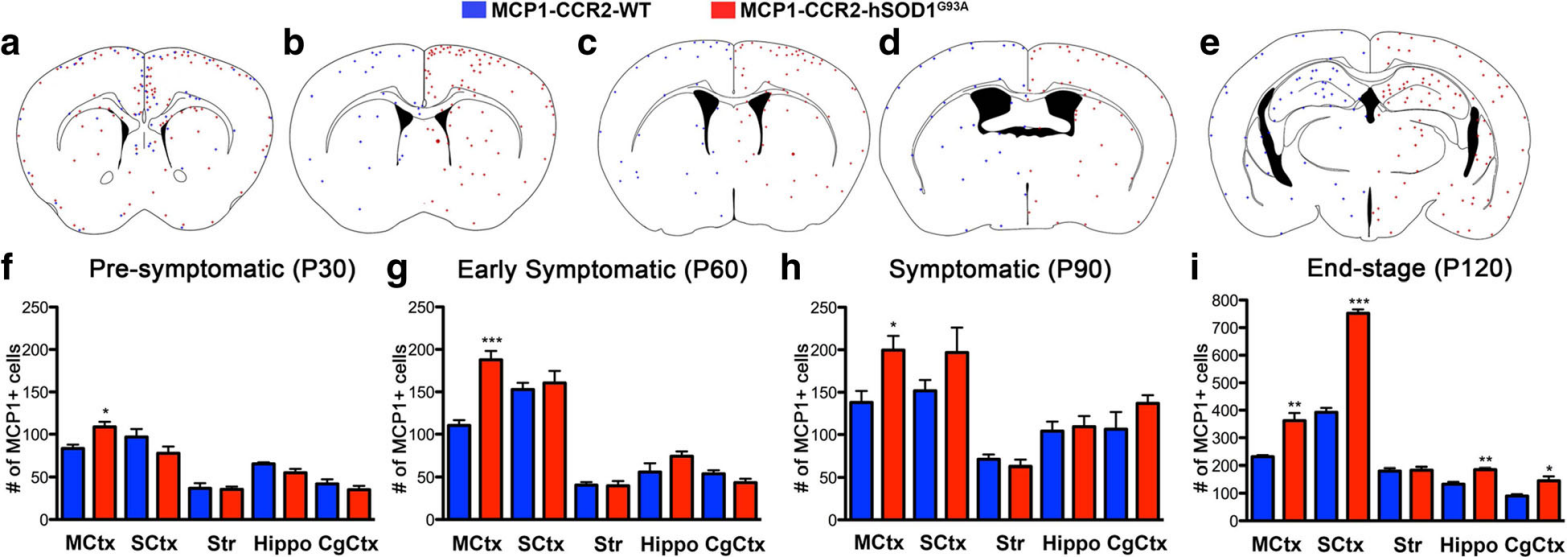
MCP1+ cells are detected early in the motor cortex of MCP1-CCR2-hSOD1^G93A^ mice. **a**-**e** Representative drawings showing the relative location of MCP1+ cells in the motor cortex, somatosensory cortex, striatum, hippocampus, and cingulate cortex within the five brain sections studied. **f**-**h** *Graphs* represent average number of MCP1+ cells per area of interest ± SEM in MCP1-CCR2-WT mice (*blue columns*) and MCP1-CCR2-hSOD1^G93A^ mice (*red column*) at the pre-symptomatic (P30), early symptomatic (P60), symptomatic (P90), and end stage (P120). Increased numbers of MCP1+ cells are detected in the primary motor cortex of MCP1-CCR2-hSOD1^G93A^ mice as early as P30. Abbreviations: *MCtx* motor cortex, *SCtx* somatosensory cortex, *Str* striatum, *Hippo* hippocampus, *CgCtx* cingulate cortex. *p < 0.05, **p < 0.01, ***p < 0.01; MCP1-CCR2-WT mice vs. MCP1-CCR2-hSOD1^G93A^ mice

To investigate whether an innate immune response occurs selectively in one region of the brain, or if there is a wide spread response at different stages of the disease, we next studied the extent of MCP1+ cells in different regions of the brain including the motor cortex. Five comparable coronal sections encompassing the motor cortex (MCtx), somatosensory cortex (SCtx), striatum (Str), hippocampus (Hippo), and cingulate cortex (CgCtx) were analyzed in both MCP1-CCR2-WT and MCP1-CCR2-hSOD1^G93A^ mice (Fig. 6a–e). Distribution of MCP1+ cells throughout the different areas of the brain including the sensory-motor cortex and striatum was very low in the MCP1-CCR2-WT mice. In contrast, increased numbers of MCP1+ cells were evident in the motor cortex of MCP1-CCR2-hSOD1^G93A^ mice starting at P30 (MCP1-CCR2-WT: 83 ± 5 vs. MCP1-CCR2-hSOD1^G93A^: 109 ± 6, *p* = 0.0101, Fig. 6f) and their numbers remained high throughout disease onset and progression (P60: MCP1-CCR2-WT: 110 ± 6 vs. MCP1-CCR2-hSOD1^G93A^: 188 ± 11, *p* = 0.0007, Fig. 6g; P90: MCP1-CCR2-WT: 138 ± 14 vs. MCP1-CCR2-hSOD1^G93A^: 200 ± 17, *p* = 0.0459, Fig. 6h; and P120: MCP1-CCR2-WT: 231 ± 6 vs. MCP1-CCR2-hSOD1^G93A^: 362 ± 27, *p* = 0.0095, Fig. 6i). In the somatosensory cortex, however, MCP1+ cells were increased only at end stage (P30: MCP1-CCR2-WT: 97 ± 9 vs. MCP1-CCR2-hSOD1^G93A^: 78 ± 8, Fig. 6f; P60: MCP1-CCR2-WT: 153 ± 8 vs. MCP1-CCR2-hSOD1^G93A^: 161 ± 14, Fig. 6g; P90: MCP1-CCR2-WT: 152 ± 13 vs. MCP1-CCR2-hSOD1^G93A^: 197 ± 29, Fig. 6h; and P120: MCP1-CCR2-WT: 392 ± 17 vs. MCP1-CCR2-hSOD1^G93A^: 752 ± 14, *p* < 0.0001, Fig. 6i). Of note, numbers of MCP1+ cells were comparable in the striatum, where axonal tracts of subcerebral projection neurons, including CSMN, are present (P30: MCP1-CCR2-WT: 37 ± 6 vs. MCP1-CCR2-hSOD1^G93A^: 36 ± 3, Fig. 6f; P60: MCP1-CCR2-WT: 41 ± 4 vs. MCP1-CCR2-hSOD1^G93A^: 40 ± 6, Fig. 6g; P90: MCP1-CCR2-WT: 71 ± 6 vs. MCP1-CCR2-hSOD1^G93A^: 63 ± 8, Fig. 6h; and P120: MCP1-CCR2-WT: 181 ± 9 vs. MCP1-CCR2-hSOD1^G93A^: 184 ± 12, Fig. 6i). The number of MCP1+ cells in the hippocampus and cingulate cortex were relatively low in both MCP1-CCR2-WT and MCP1-CCR2-hSOD1^G93A^ mice and increased only during end stage (P30: Hippo, MCP1-CCR2-WT: 65 ± 2 vs. MCP1-CCR2-hSOD1^G93A^: 55 ± 5; CgCtx, MCP1-CCR2-WT: 42 ± 5 vs. MCP1-CCR2-hSOD1^G93A^: 35 ± 5, Fig. 6f; P60: Hippo, MCP1-CCR2-WT: 56 ± 10 vs. MCP1-CCR2-hSOD1^G93A^: 74 ± 6; CgCtx, MCP1-CCR2-WT: 54 ± 4 vs. MCP1-CCR2-hSOD1^G93A^: 44 ± 5, Fig. 6g; P90: Hippo, MCP1-CCR2-WT: 104 ± 11 vs. MCP1-CCR2-hSOD1^G93A^: 109 ± 12; CgCtx, MCP1-CCR2-WT: 106 ± 20 vs. MCP1-CCR2-hSOD1^G93A^: 137 ± 9, Fig. 6h; and P120: Hippo, MCP1-CCR2-WT: 133 ± 8 vs. MCP1-CCR2-hSOD1^G93A^: 186 ± 6, *p* = 0.0057; CgCtx, MCP1-CCR2-WT: 90 ± 6 vs. MCP1-CCR2-hSOD1^G93A^: 145 ± 16, *p* = 0.0307, Fig. 6i). Altogether, these results suggest the presence of a distinct pattern for the origination and propagation of innate immunity in the cerebral cortex of hSOD1^G93A^ mice and point to an early increase of MCP1+ cells, especially in the motor cortex, where they might play a key role.

### Characterization of MCP1+ and CCR2+ cells in the motor cortex

**Fig. 7:**
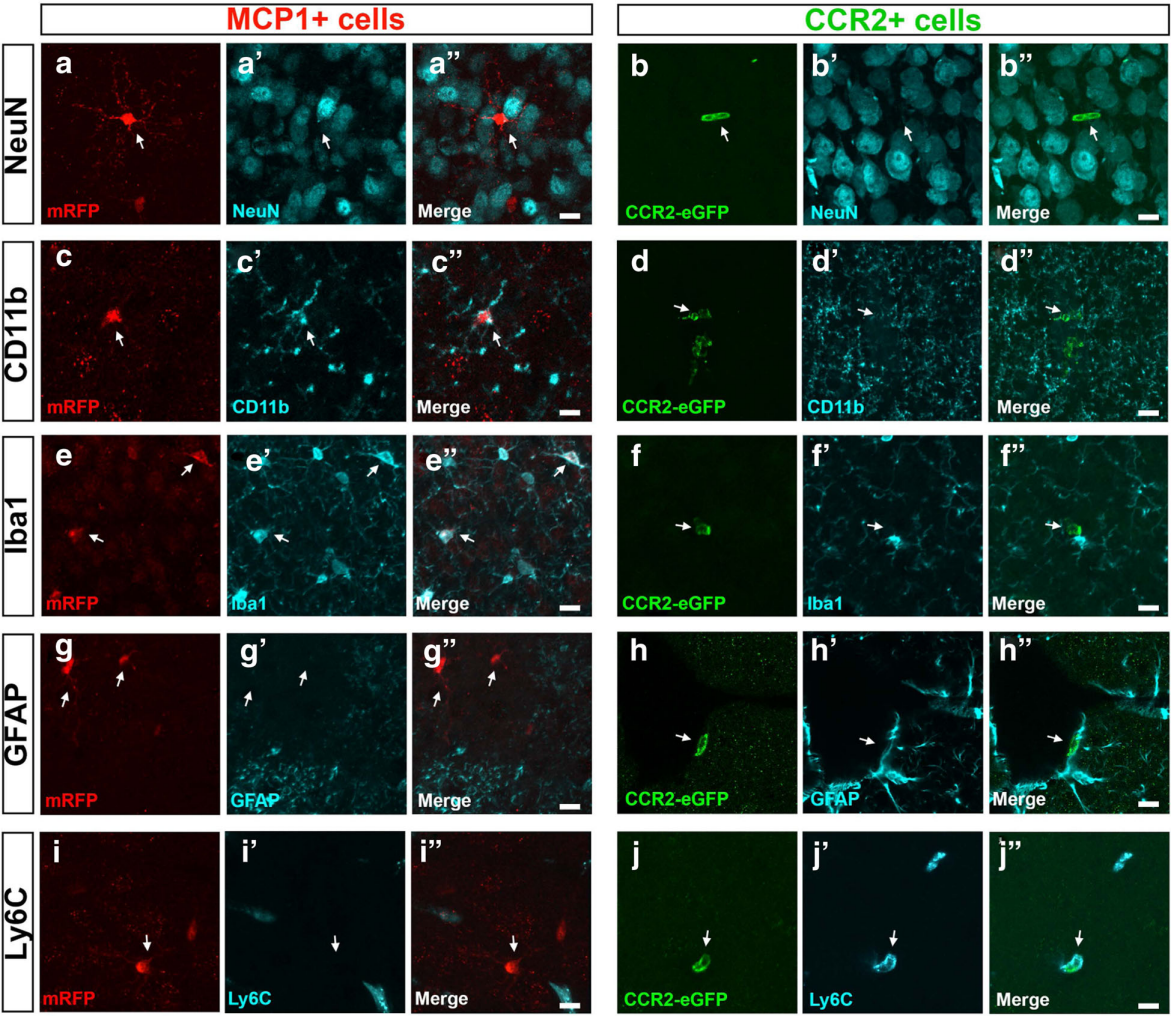
MCP1+ cells are mainly microglia and some CCR2+ cells are infiltrating monocytes in the motor cortex. **a**, **b** Representative images of immunohistochemistry for neuronal marker NeuN, MCP1+ cells (**a**, **a’**, **a”**), and CCR2+ cells (**b**, **b’**, **b”**) reveal that neither MCP1+ nor CCR2+ cells are mature neurons. **c**, **e** Representative images of immunohistochemistry for monocyte/macrophage lineage marker CD11b (**c**, **c’**, **c”**), microglia marker Iba1 (**e**, **e’**, **e”**), and MCP1+ cells demonstrate co-localization and microglial lineage. **d**, **f** Representative images of immunohistochemistry for microglia/monocyte lineage marker CD11b (**d**, **d’**, **d”**), microglia marker Iba1 (**f**, **f’**, **f”**), and CCR2+ cells show close cellular interaction but lack of co-localization with microglia markers. **g**, **i** Representative images of immunohistochemistry for astrocyte (GFAP+: **g**, **g’**, **g”**) and infiltrating monocyte (Ly6C+: **i**, **i’**, **i”**) markers reveal lack of co-localization with MCP1+ cells. **h**, **j** Representative images of immunohistochemistry for astrocyte (GFAP+: **h**, **h’**, **h”**) and infiltrating monocyte (Ly6C+: **j**, **j’**, **j”**) markers reveal that CCR2+ cells are in close contact with astrocytes (GFAP+) but do not express GFAP themselves. However, a subpopulation of CCR2+ cells co-localize with infiltrating monocyte marker (Ly6C). *Arrows* point to the cells of interest. *Scale bar* = 20 μm

We next investigated the cellular identity of MCP1+ and CCR2+ cells in the motor cortex. For this purpose, we evaluated co-localization of MPC1+ or CCR2+ with specific cellular markers for neurons (NeuN+), astrocytes (GFAP+), microglia (Iba1+, CD11b+), and infiltrating monocytes (Ly6C+). These analyses were performed at the symptomatic stage (P60) when high numbers of both MCP1+ and CCR2+ cells were mostly observed both in the motor cortex and spinal cord. None of the MCP1+ cells (0%, *N* = 1075) or CCR2+ (0%, *N* = 27) were neurons as they lacked NeuN (Fig. 7a, b) and Map2 expression (Fig. 3a–i). Since the majority of the MCP1+ cells in the spinal cord were microglia and MCP1+ cells in the motor cortex also have cellular characteristics resembling microglia with ramified thin protrusions and a small cell body, we investigated whether MCP1+ cells in the motor cortex belonged to the microglia lineage. MCP1+ cells were mainly microglia in MCP1-CCR2-hSOD1^G93A^ mice, as evidenced by co-localization with both CD11b (99 ± 1%, *N* = 1308, Fig. 7c) and Iba1 (99 ± 1%, *N* = 802, Fig. 7e). Similar results were observed in the cortex of MCP1-CCR2-WT mice (MCP1/NeuN co-localization 0%, *N* = 1096; CCR2/NeuN co-localization 0%, *N* = 32; MCP1/CD11b co-localization 99 ± 1%, *N* = 875; MCP1/Iba1 co-localization 99 ± 1%, *N* = 846). Interestingly, CCR2+ cells were not microglia (Fig. 7f), but they co-localized with monocyte/macrophage marker CD11b (Fig. 7d) and displayed close interactions with MCP1+ cells and microglia. Neither MCP1+ cells (GFAP: MCP1-CCR2-hSOD1^G93A^ 0%, *N* = 1075, Fig. 7g; MCP1-CCR2-WT, 0%, *N* = 1096) nor CCR2+ cells (GFAP: MCP1-CCR2-hSOD1^G93A^, 0%, *N* = 27, Fig. 7h; MCP1-CCR2-WT, 0% *N* = 32) were astrocytes, although direct interaction of CCR2+ cells with astrocytes was frequently observed.

Based on previous reports in ALS mouse models and studies performed in ALS patients [27, 31], we next evaluated the possibility that MCP1+ and CCR2+ cells belong to a monocyte/macrophage lineage. We hypothesize that infiltrating monocytes could express CCR2 and be recruited to the motor cortex in MCP1-CCR2-hSOD1^G93A^ mice as a response to increased levels of MCP1. A small subset of CCR2+ cells was indeed infiltrating monocytes during symptomatic stage, as they expressed Ly6C, a reliable marker (MCP1-CCR2-WT 10 ± 6%, *N* = 15; MCP1-CCR2-hSOD1^G93A^ 19 ± 5%, *N* = 21; Fig. 7j). Unlike CCR2+ cells, MCP1+ cells were not infiltrating monocytes and they did not belong to that lineage (MCP1-CCR2-hSOD1^G93A^ 0%, *N* = 499; Fig. 7i).

In summary, our results indicate that the majority of MCP1+ cells belong to a microglia lineage in both MCP1-CCR2-WT and MCP1-CCR2-hSOD1^G93A^ mice and that a subset of CCR2+ cells are infiltrating monocytes, further implicating their potential role in motor neuron pathology during disease.

### Evidence of cell-cell interactions between CSMN apical dendrites and innate immune cells

Since MCP1+ and CCR2+ cells were significantly increased in the motor cortex at P60 (Fig. 6g), we evaluated their presence and cellular characteristics in the motor cortex. Few CD11b+ MCP1 cells were detected in MCP1-CCR2-WT mice at P60 (Additional file 2: Figure S2a). Conversely, high levels of MCP1+ cells expressing CD11b were present in the motor cortex of MCP1-CCR2-hSOD1^G93A^ mice (Additional file 2: Figure S2b). In some instances, cellular interactions between CCR2+ cells and CD11b+ MCP1 cells were observed (Additional file 2: Figure S2b”’).

We previously reported apical dendrite defects of CSMN in hSOD1^G93A^ mice, which exhibited signs of degeneration and disintegration at P60 [22]. Since CSMN are mainly modulated especially in layer II/III of the motor cortex via numerous neurons, such as long-distance projecting neurons and local circuitry neurons [40], the health and integrity of their apical dendrites is of particular importance for their function.

**Fig. 8:**
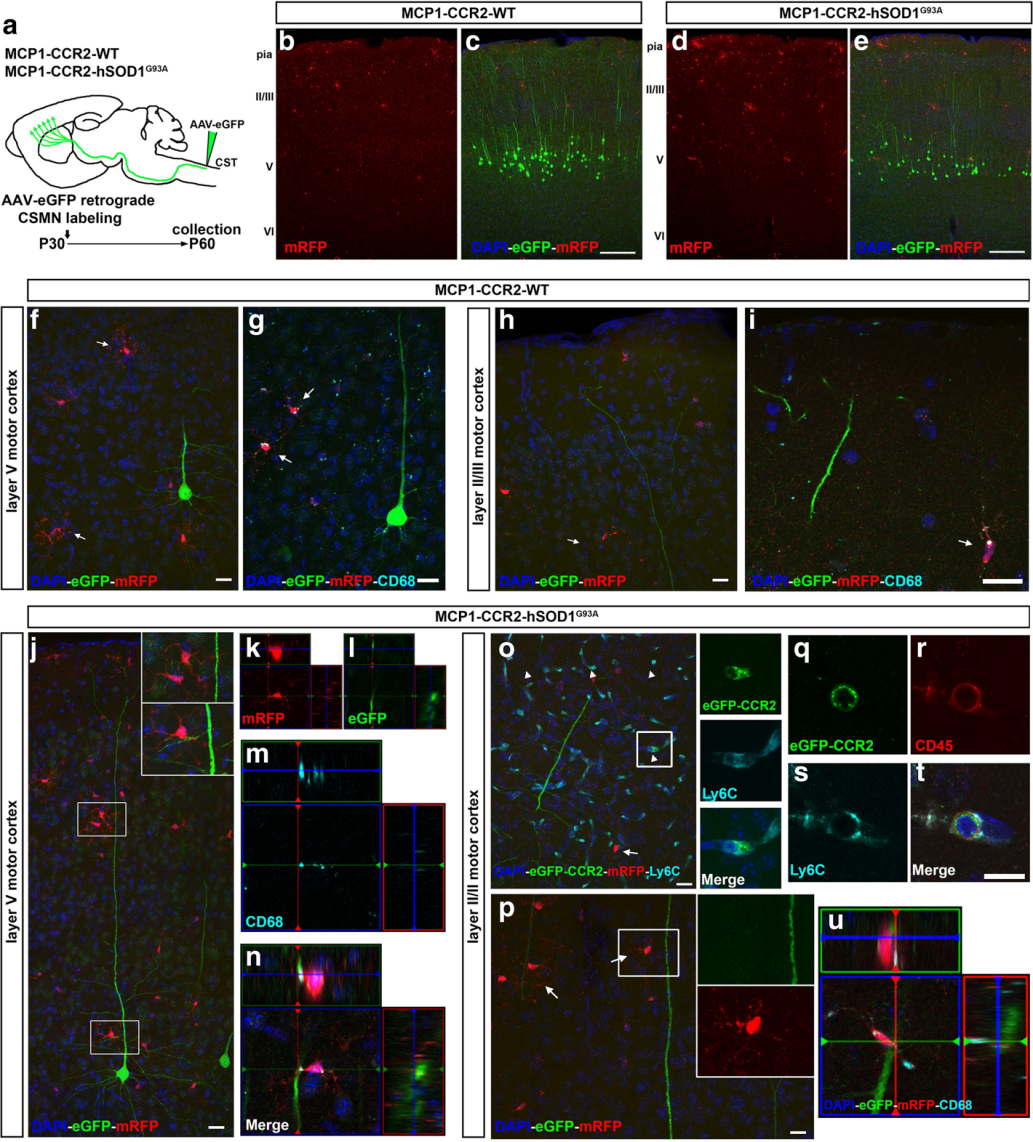
MCP1+ and CCR2+ cells are located in close proximity to CSMN in the MCP1-CCR2-hSOD1^G93A^ mice. **a** Experimental design depicting retrograde transduction of CSMN approach using AAV-eGFP in the MCP1-CCR2-WT and MCP1-CCR2-hSOD1^G93A^ mice. AAV2-eGFP was injected into the CST of mice at P30, and tissue was collected at P60. **b**, **c** Representative image shows relative location of MCP1+ cells and transduced CSMN (eGFP+) in the motor cortex of MCP1-CCR2-WT mice. **d**, **e** Representative image shows relative location and increased numbers of MCP1+ cells and transduced CSMN (eGFP+) in the motor cortex of MCP1-CCR2-hSOD1^G93A^ mice. **f**–**i** Representative images showing MCP1+ cells that co-localize with phagocytic marker CD68 and their location in respect to transduced CSMN (eGFP+) in the motor cortex of MCP1-CCR2-WT mice. MCP1+ cells have ramified morphology with low levels of CD68, and there is no evidence of direct interaction with healthy CSMN neither at the layer V (**f**, **g**)—cell body—nor at the layer II/III (**h**, **i**), apical dendrite level. **j** Representative image shows MCP1+ cells in close proximity of diseased CSMN (**j**, *insets*) in the layer V of motor cortex in MCP1-CCR2-hSOD1^G93A^ mice. **k**–**n** Orthogonal views demonstrate cell-cell interactions of MCP1+ cells expressing phagocytic marker CD68 and diseased CSMN in the layer V of motor cortex demonstrate cell-cell interactions with diseased CSMN in the MCP1-CCR2-hSOD1^G93A^ mice. **o**–**u** Representative images showing interactions of MCP1+ and CCR2+ cells with apical dendrites of diseased CSMN in the layer II/III of motor cortex in MCP1-CCR2-hSOD1^G93A^ mice. **o** Representative image showing CCR2+ cells in close proximity to the apical dendrite of a diseased CSMN in MCP1-CCR2-hSOD1^G93A^ mice. CCR2+ cells co-localize with infiltrating monocyte marker Ly6C (**o**, *arrowhead* and *insets* enlarged to the right) and monocyte CD45 (**q**–**t**). **p** Apical dendrites are vacuolated and surrounded by several MCP1+ cells (*Insets* are enlarged to the right). **u** Orthogonal view of direct cell-cell interactions of MCP1+ cell expressing phagocytic marker CD68 with vacuolated dendrite of diseased CSMN in the motor cortex of MCP1-CCR2-hSOD1^G93A^ mice. *Scale bars*: **b**–**e** = 200 μm; **f**–**j**, **o**, **p** = 20 μm; **q**–**t** = 10 μm

In an effort to visualize and assess cellular interactions between MCP1+ cells, CCR2+ cells, and CSMN, we used an AAV-mediated retrograde labeling approach [22] to transduce and visualize CSMN with eGFP expression in the motor cortex of MCP1-CCR2-WT and MCP1-CCR2-hSOD1^G93A^ mice (Fig. 8a). Low-magnification images show overall distribution of MCP1+ cells in relation to CSMN in the motor cortex of MCP1-CCR2-WT (Fig. 8b, c), and as expected (Fig. 6g), MCP1+ cells were more prominent in the motor cortex of MCP1-CCR2-hSOD1^G93A^ mice (Fig. 8d, e). CSMN had healthy cell bodies and apical dendrites in the MCP1-CCR2-WT mice and few MCP1+ cells were observed in their vicinity, which displayed cellular characteristics of resting microglia with thin processes and small cell bodies (Fig. 8f, g; arrows). MCP1+ cells also expressed low levels of phagocytic marker CD68 in the MCP1-CCR2-WT mice (Fig. 8g; arrows). MCP1+ cells were also present in the layer II/III of the motor cortex in the MCP1-CCR2-WT mice (Fig. 8h, i; arrows), and there was a lack of direct interaction of MCP1+ cells expressing CD68 with CSMN apical dendrites (Fig. 8i, arrow). In contrast, CSMN of MCP1-CCR2-hSOD1^G93A^ mice displayed different levels of vacuolation in the proximal and distal apical dendrites (Fig. 8j–u). CSMN apical dendrites with vacuoles were surrounded by several MCP1+ cells in close proximity to either at their cell body (Fig. 8j; Additional file 2: Figure S2d-e) or apical dendrite (Fig. 8o, p; Additional file 2: Figure S2f-g). There was also evidence of direct cell-cell interactions especially at the site of degenerating apical dendrites (Fig. 8j, insets). To further demonstrate cell-cell interactions of MCP1+ cells with CSMN, orthogonal views from z-stacks were evaluated. Direct interaction of MCP1+ cells expressing CD68 marker with CSMN was observed, and in some cases, the thin protrusions of MCP1+ cells were wrapping around CSMN apical dendrites in the layer V (Fig. 8k–n; Additional file 2: Figure S2h-j) and in the layers II/III (Fig. 8u) of the motor cortex in the MCP1-CCR2-hSOD1^G93A^ mice. We further evaluated the phenotype of these MCP1+ microglia (Additional file 3: Figure S3) using known functional markers for beneficial/M2 microglia (Arginase 1, Arg1, Additional file 3: Figure S3a-b) or detrimental/M1 microglia (inducible nitric oxide synthase, iNOS, Additional file 3: Figure S3c-d) [15, 41]. Different from previously published reports in the hSOD1^G93A^ spinal cord [42], neither Arg1 (Additional file 3: Figure S3f-g) nor iNOS (Additional file 3: Figure S3h-i) was detected in MCP1+ microglia in the MCP1-CCR2-WT or MCP1-CCR2-hSOD1^G93A^ motor cortex. Finally, a subset of CCR2+ cells that co-localize with infiltrating monocyte marker Ly6C were also detected juxtaposed to the degenerating CSMN apical dendrites (Fig. 8o, insets). The monocyte nature of these CCR2+ cells expressing Ly6C was also demonstrated by co-localization with monocyte marker CD45 [28] (Fig. 8q–t; Additional file 3: Figure S3k-n). Altogether, our results suggest a possible role of MCP1+ cells in debris clearance from CSMN since MCP1+ cells expressing CD68 were found wrapping around degenerating CSMN apical dendrites and support the presence of infiltrating monocytes in the motor cortex of MCP1-CCR2-hSOD1^G93A^ mice.

### Evidence of cell-cell interactions between Betz cell apical dendrites and innate immune cells in ALS patients

**Fig. 9:**
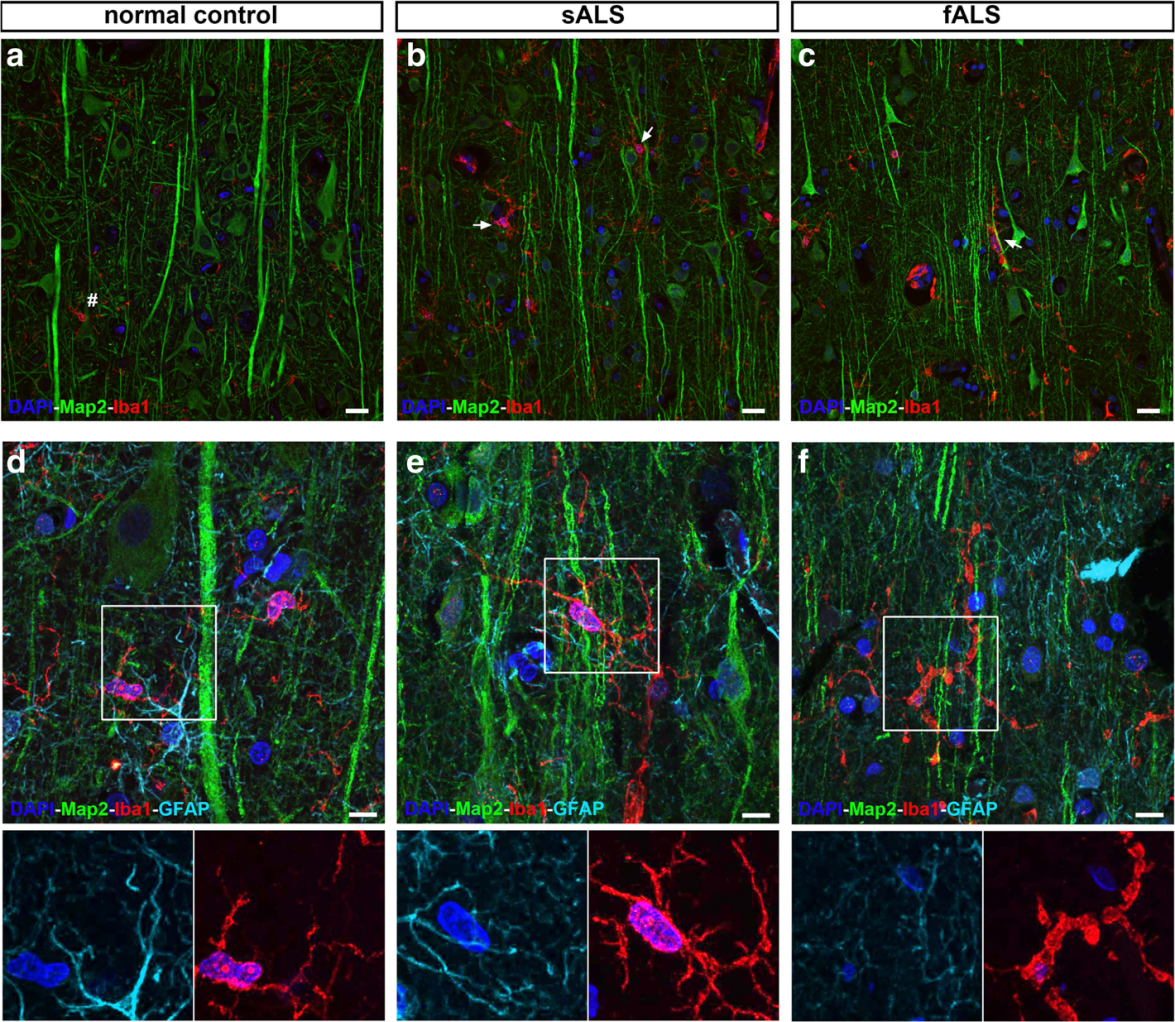
Microgliosis is observed near degenerating apical dendrites of Betz cells in motor cortex of ALS patients. Representative images of apical dendrites (Map2+) located in layer IV of the motor cortex isolated from normal controls and ALS patients. **a**, **d** Apical dendrites appear healthy in normal controls, and there is no evidence of gliosis. *Insets* are enlarged at the bottom. **b**–**f** Vacuolated apical dendrites with obvious structural defects are present in both sALS (**b**, **e**) and fALS (**c**, **f**) cases with microgliosis (Iba1+) and astrogliosis (GFAP+). In some cases, activated microglia are in contact with degenerating apical dendrites (**c**, *arrow* and **e**, **f**), and abnormal rod-like microglia are also observed (**f**). *Insets* are enlarged at the bottom. *Number sign* marks normal microglia; *arrows* point to activated microglia. Abbreviations: *sALS* sporadic ALS, *fALS* familial ALS. *Scale bar* = 20 μm (**a**–**c**), *scale bar* = 10 μm (**d**–**f**).

We recently reported that Betz cells of a broad spectrum of ALS patients display major structural defects especially in their apical dendrites, which include vacuoles of different sizes and numbers, as well as aberrant signs of disintegration [43]. In line with our findings of MCP1+ cells present in close proximity and displaying cell-cell contact with diseased CSMN apical dendrites in MCP1-CCR2-hSOD1^G93A^ mice, many of the ALS patient Betz cells also had MCP1+ cells in close proximity and at times aligned in line with apical dendrites (Fig. 1d–g). In normal controls, Betz cells had healthy apical dendrites and no signs of microgliosis was observed (Fig. 9a, d, insets). Conversely, in sALS and fALS subjects, Betz cells had numerous vacuoles ranging in size with activated microglia in their close vicinity (Fig. 9b, c, e, f, insets) and abnormal rod-like microglia were present (Fig. 9f, insets). These findings not only reaffirm recapitulation of findings in human but also strengthen the idea that focusing on cells and neurons of interest will be translational, and that MCP1-CCR2-hSOD1^G93A^ mice offer a great tool to study and understand the cellular and molecular basis of the interplay between vulnerable motor neurons of patients and the cells of the innate immune response.

## Discussion

Visualization of an innate immune response in a model of ALS overcomes many current limitations in the field. By examining the expression pattern of MCP1, a pivotal chemokine that has been detected in the CSF and serum of ALS patients and suggested to play a central role in neuroinflammation and neurodegeneration [23, 24, 31, 44, 45], we have started to define the specific components of the innate immune response in ALS cortex and spinal cord at a cellular level. To this end, we have generated and characterized a novel ALS reporter line, in which cells that express MCP1 and CCR2 are detected by an intrinsic fluorescent gene expression, in vivo.

Neuroinflammatory response is observed in both ALS patients and mouse models of ALS [2, 38], and developing evidence suggests a critical role for MCP1/CCR2 axis in ALS pathology [27, 31, 46]. MCP1 is normally expressed at very low levels in CNS but is detected at high levels in glia and macrophages in spinal cords of ALS patients [23], and it is also detected in the CSF and blood plasma of ALS patients [23–25, 31]. Early reports suggested correlation between the levels of circulating monocytes expressing CCR2 and development of ALS [31, 32]. Recent studies reinforced this notion by reporting dysregulation of “classical” vs. “non-classical” circulating monocytes that are skewed towards a proinflammatory profile in ALS patients [29, 30], further demonstrating that these cells were reduced in the plasma as they were recruited to the CNS. Clinical studies have also correlated MCP1/CCR2 levels with ALS symptoms and progression, reporting decreased levels of peripheral mononuclear cells expressing CCR2 in ALS patients especially with limb onset [47] and positive correlation between increased expression of MCP1 and ALS symptoms [48]. Altogether, these studies suggest the importance of MCP1/CCR2 pathway as regulator of monocyte chemotaxis towards sites of neurodegeneration in ALS.

The recruitment of monocytes into areas of the CNS where neurodegeneration occurs in ALS is currently under scrutiny [27, 31, 32]. Our understanding of the neuroinflammatory response in ALS relies heavily upon findings from ALS mouse models, particularly the hSOD1^G93A^ mice, which recapitulate most of the clinical manifestations observed in patients, including increased neuroinflammation especially in the spinal cord. Thus, in hSOD1^G93A^ mice, the recruitment and increase of Ly6C+ CCR2 cells to the spinal cord during disease progression was reported, associated with increased of MCP1 by resident microglia [27]. However, other studies have reported barely detectable monocyte recruitment into the spinal cord parenchyma up to progressive stages of the disease [49] or modest monocyte recruitment with high percentage of T cell marker expression (TCRβ+) in the hSOD1^G93A^ mice [28]. Previous studies were more focused on assessing recruitment of Ly6C+ monocytes to the spinal cord and not to the brain in the hSOD1^G93A^ mice. In our studies, we report a modest presence of CCR2+ cells expressing infiltrating monocyte marker Ly6C, particularly in small blood vessels in the motor cortex. Since CCR2+ cells are low in numbers in the motor cortex of hSOD1^G93A^ mice, it is possible that previous studies were not able detect their presence in the motor cortex since they relied upon flow cytometry analysis which might require a larger number of cells to detect changes.

Our findings reveal that the vast majority of MCP1+ cells are microglia. Interestingly, MPC1+ microglia in the spinal cord and motor cortex of MCP1-CCR2-hSOD1^G93A^ mice had different morphology. In the spinal cord, MCP1+ microglia displayed an activated state morphology characterized by short protrusions and hypertrophy early in the disease that was accompanied by a dramatic increment in their numbers (91% increase) in comparison to those of the motor cortex (42%). These observations are in agreement with the strong microgliosis previously reported in the in the spinal cord of the hSOD1^G93A^ mice, and it supports the idea that MCP1+ microglia has morphological characteristics of M1 microglia phenotype in the MCP1-CCR2-hSOD1^G93A^ mice [11, 15, 39, 42]. Although, accumulation of MCP1+ microglia in the motor cortex was more modest they also continue to increase during disease progression. Interestingly, there was evidence of cell-cell interaction of vulnerable CSMN with MCP1+ and CCR2+ cells, detected both along the apical dendrites and soma, suggesting that such intricate interaction could indeed be a potential contributor to CSMN pathology. These MCP1+ microglia had different morphology compared to that of the spinal cord with smaller size and thin protrusions. However, MCP1+ microglia expressed phagocytic marker CD68 [50], which has recently been associated with a protective role in activated microglia in ALS [51]. Therefore, our results suggest that MCP1+ microglia, especially the ones wrapping around the CSMN apical dendrites that display vacuolation might have a protective role by clearing debris and assessing CSMN health in the motor cortex during disease. In support of this idea, the cellular analyses of postmortem human samples used in this study reveals Iba1+ activated microglia in contact with vacuolated Betz dendrites, with increased levels of MCP1 in the motor cortex of patients with sALS and fALS and further reinforce the importance of generating and characterizing MCP1-CCR2-hSOD1^G93A^ mice.

Previous studies of MCP1/CCR2 axis mostly relied upon qRT-PCR, western blots, flow cytometry, and immunocytochemistry to investigate the identity of cells expressing MCP1 and CCR2 [23, 26, 52]. MCP1-CCR2-hSOD1^G93A^ mice offer great advantages for detailed cellular analysis and cell-cell interaction studies in vivo. In addition, MCP1+ and CCR2+ cells can be purified using fluorescent activated cell sorting (FACS) approaches, allowing further molecular and genetic analysis, which may reveal the underlying genetic signature of these cells and how that changes over time and with respect to location in CNS. In addition, crossbreeding MCP1-CCR2 mice with other well-defined disease models can now generate numerous other disease reporter lines. The MCP1+ and CCR2+ cells in other disease models may express a different set of genes and secrete a different set of cytokines and chemokines. The common and unique aspects of these factors would help us understand the converging and diverging paths that lead to differential motor neuron vulnerability in different cases of ALS and other motor neuron diseases.

Detailed cellular analysis of the MCP1/CCR2 axis may reveal novel targets for therapeutic interventions and may help develop better clinical trials that aim to modulate the purely deleterious aspects of the innate immune response during disease progression. To date a number of clinical trials have aimed at modulating the entire immune response, but they represented studies in which both the beneficial and detrimental effects of innate immunity were simultaneously eliminated and thus resulted in mixed and inconclusive results. For example, the clinical trials with anti-inflammatory agents such as minocycline [53], thalidomide [54], and celecoxib [55] all failed in phase II or III. However, we need to acknowledge that none of these compounds were specifically targeting a known and well-documented inflammatory pathway in ALS and that a heterogeneous patient population was recruited to the study. For instance, NP001 is a compound targeting macrophage activation that shows more promise in clinical trials [56]. Moreover, recent findings report that ALS patients with elevated levels of C-reactive protein in their serum respond better to NP001 [57]. This illustrates that using more specific therapeutic approaches such as targeting neuroinflammation in patients with an elevated neuroinflammatory state is a better therapeutic strategy. It seems likely that the success rate of clinical trials can be improved if the detailed underlying cellular nature of the innate immune response and details of the motor neuron-immune axis in ALS is elucidated. It is possible that the underlying basis of immune reactions will be different in the brain and the spinal cord and that cells which constitute innate immunity will either be recruited differently or will display distinct modes of pathology.

## Conclusions

In summary, our results help us visualize the intricate balance between innate immunity and motor neuron vulnerability by bringing cellular clarity to their interaction both in the spinal cord and the motor cortex. Potential importance for microglia in the motor cortex is supported by clinical studies demonstrating rapid disease progression and microglia pathology [18–21]. Therefore, studying the motor neuron circuitry at different levels would help reveal potentially different treatment strategies for patients who manifest the disease first in the motor cortex or in the spinal cord. Revealing a correlation between the timing and the extent of neuron loss with the cells, molecules, cytokines, and chemokines of immune response will be transformational. Recapitulation of findings in human ALS patients suggest close correlation and potential impact for identifying novel targets and for building better and more affective clinical trials for effective and log-term treatments of ALS and other diseases in which upper motor neurons are impaired.

## Additional files

Additional file 1: Figure S1. Immunohistochemistry analysis confirms that mRFP+ cells express MCP1 in MCP1::mRFP transcription reported mice. (a-f) Representative images of mRFP+ cells (red) that co-localize with MCP1 protein (green) in the motor cortex of MCP1-CCR2-WT mice. Scale bar = 20 μm. (PDF 172 kb)

Additional file 2: Figure S2. MCP1+ cells and CCR2+ cells are present in the motor cortex and they are in close proximity to CSMN in the MCP1-CCR2-hSOD1^G93A^ mice. (a) Representative image showing very few MCP1+ cells in the motor cortex of MCP1-CCR2-WT mice. (b) Representative image showing increased numbers of MCP1+ cells in the motor cortex of MCP1-CCR2-hSOD1^G93A^ mice. MCP1+ cells express microglia marker CD11b. Insets enlarged to the right (b’-b”’). (c) Experimental design depicting retrograde transduction of CSMN approach using AAV-eGFP in the MCP1-CCR2-WT and MCP1-CCR2-hSOD1^G93A^ mice. AAV2-eGFP was injected into the CST of mice at P30, and tissue was collected at P60. (d, e) Representative images show MCP1+ cells near transduced CSMN (eGFP+) in the layer V of the motor cortex (d, e) and in the layer II/III of the motor cortex (f, g) in MCP1-CCR2-hSOD1^G93A^ mice. (h, j) Representative images show MCP1+ cells expressing phagocytic marker CD68 and their interaction with transduced CSMN in the layer V of motor cortex in the MCP1-CCR2-hSOD1^G93A^ mice. (k-n) Representative image showing CCR2+ cells in layer II/III of motor cortex co-localizing with monocyte marker CD45 and infiltrating monocyte marker Ly6C. Scale bar:s: a,b,d-g =20 μm; k-n = 10 μm. (PDF 1521 kb)

Additional file 3: Figure S3. MCP1+ cells express neither Arginase 1 (Arg1) nor inducible nitric oxide synthase (iNOS) in the MCP1-CCR2-hSOD1^G93A^ mice. (a) Representative images of Arg1+ cells (arrowheads) and MCP1+ cells (arrows) in the liver of MCP1-CCR2- hSOD1^G93A^ mice 6 h post LPS I.P. injection (positive control). (b) Representative images of 2° only for Arg1 (negative control) and MCP1+ cells (arrows) in the liver of MCP1-CCR2- hSOD1^G93A^ mice 6 h post LPS I.P. injection. (c) Representative images of MCP1+ cells (arrows) in the spleen of MCP1-CCR2- hSOD1^G93A^ mice 6 h post LPS I.P. injection (positive control) show co-localization with iNOS (arrows). (d) Representative images of 2° only for iNOS (negative control) and MCP1+ cells (arrows) in the spleen of MCP1-CCR2- hSOD1^G93A^ mice 6 h post LPS I.P. injection. (e) Experimental design depicting retrograde transduction of CSMN approach using AAV-eGFP in the MCP1-CCR2-WT and MCP1-CCR2-hSOD1^G93A^ mice. AAV2-eGFP was injected into the CST of mice at P30, and tissue was collected at P60. (f-g) Representative images of the layer II/III of motor cortex show lack of co-localization of MCP1+ cells with Arg1 in MCP1-CCR2-WT mice (f) and MCP1-CCR2- hSOD1^G93A^ mice (g). (h-i) Representative images of the layer II/III of motor cortex show lack of co-localization of MCP1+ cells with iNOS in MCP1-CCR2-WT mice (h) and MCP1-CCR2- hSOD1^G93A^ mice (i). Scale bar = 10 μm. (PDF 961 kb)

## Abbreviations

AAV: Adeno-associated virus
ALS: Amyotrophic lateral sclerosis
Arg1: Arginase 1
CCR2: CC chemokine receptor 2
CgCtx: Cingulate cortex
CNS: Central nervous system
CSMN: Corticospinal motor neurons
CST: Corticospinal tract
DGM: Dorsal grey matter
eGFP: Enhanced green fluorescent protein
fALS: Familial ALS
Hippo: Hippocampus
iNOS: Inducible nitric oxide synthase
LPS: Lipopolysaccharide
MCP1: Monocyte chemoattractant protein-1
Mctx: Motor cortex
PFA: Paraformaldehyde
RFP: Monomeric red fluorescent protein-1
sALS: Sporadic ALS
SCtx: Somatosensory cortex
SMN: Spinal motor neurons
Str: Striatum
VGM: Ventral gray matter
