# Supplementary figures and images for: Evidence for an early innate immune response in the motor cortex of ALS
Source: J Neuroinflammation. 2017 Jun 26;14:129. doi: 10.1186/s12974-017-0896-4 (PMC5485686; doi:10.1186/s12974-017-0896-4)

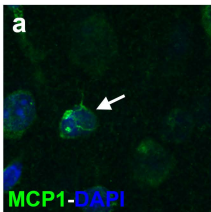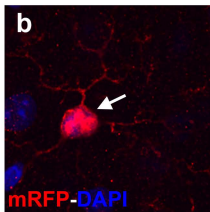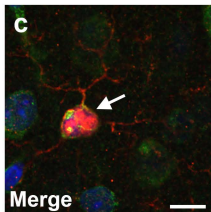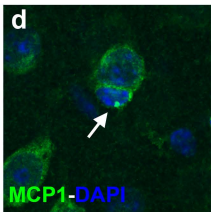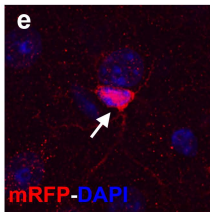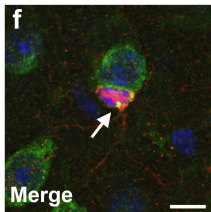

Supplement: Supplementary file 1 — Immunohistochemistry analysis confirms that mRFP+ cells express MCP1 in MCP1::mRFP transcription reported mice. (a-f) Representative images of mRFP+ cells (red) that co-localize with MCP1 protein (green) in the motor cortex of MCP1-CCR2-WT mice. Scale bar = 20 μm. (PDF 172 kb) [file 12974_2017_896_MOESM1_ESM.pdf]

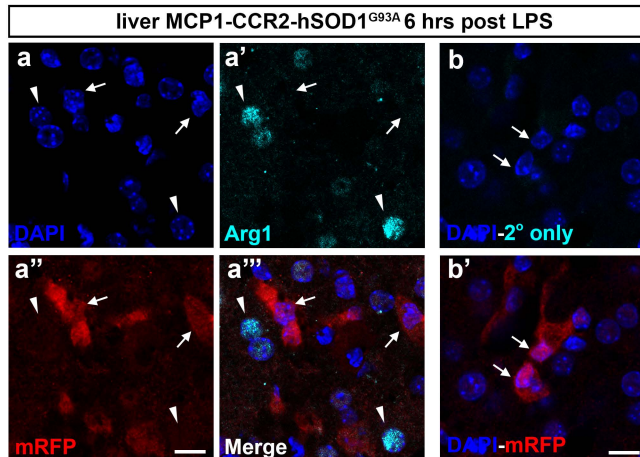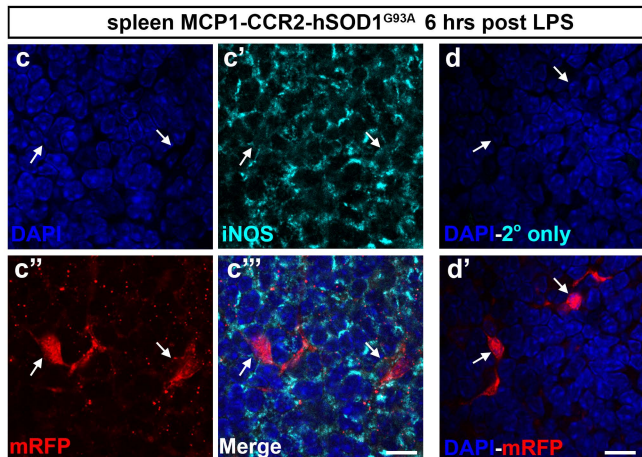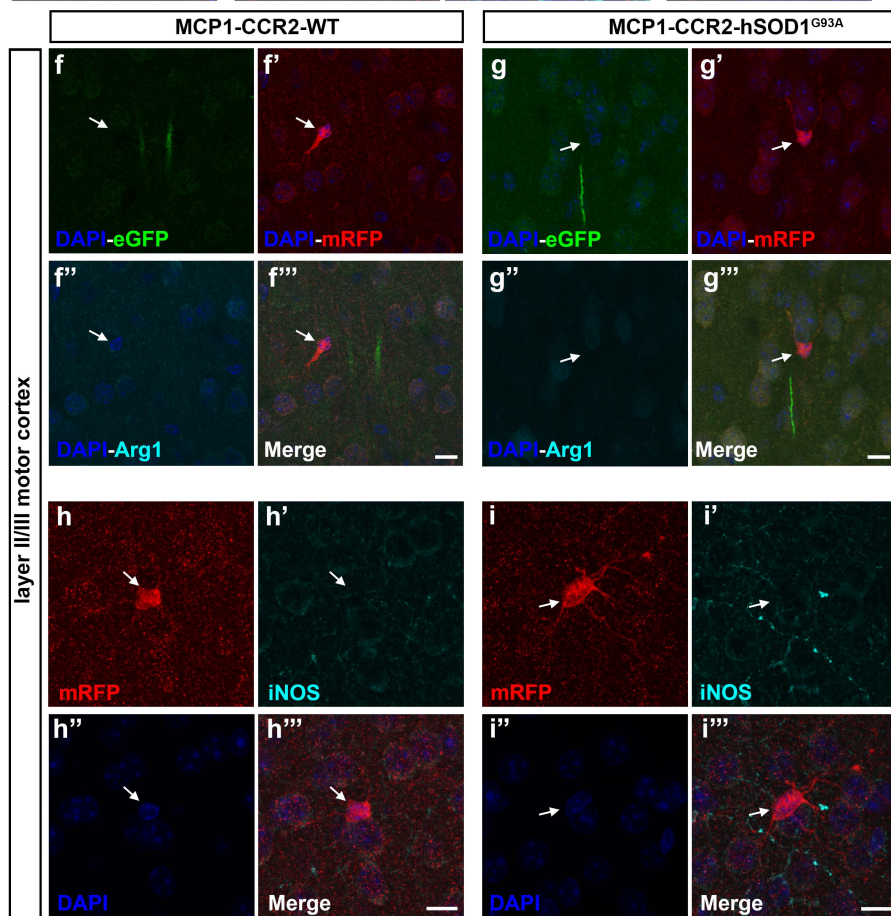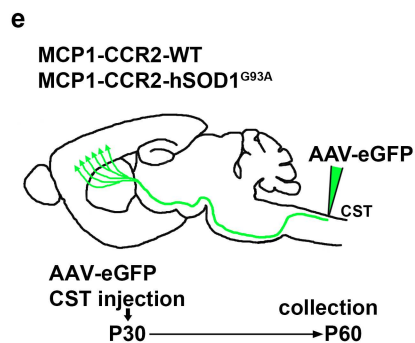

Supplement: Supplementary file 3 — MCP1+ cells express neither Arginase 1 (Arg1) nor inducible nitric oxide synthase (iNOS) in the MCP1-CCR2-hSOD1G93A mice. (a) Representative images of Arg1+ cells (arrowheads) and MCP1+ cells (arrows) in the liver of MCP1-CCR2- hSOD1G93A mice 6 h post LPS I.P. injection (positive control). (b) Representative images of 2° only for Arg1 (negative control) and MCP1+ cells (arrows) in the liver of MCP1-CCR2- hSOD1G93A mice 6 h post LPS I.P. injection. (c) Representative images of MCP1+ cells (arrows) in the spleen of MCP1-CCR2- hSOD1G93A mice 6 h post LPS I.P. injection (positive control) show co-localization with iNOS (arrows). (d) Representative images of 2° only for iNOS (negative control) and MCP1+ cells (arrows) in the spleen of MCP1-CCR2- hSOD1G93A mice 6 h post LPS I.P. injection. (e) Experimental design depicting retrograde transduction of CSMN approach using AAV-eGFP in the MCP1-CCR2-WT and MCP1-CCR2-hSOD1G93A mice. AAV2-eGFP was injected into the CST of mice at P30, and tissue was collected at P60. (f-g) Representative images of the layer II/III of motor cortex show lack of co-localization of MCP1+ cells with Arg1 in MCP1-CCR2-WT mice (f) and MCP1-CCR2- hSOD1G93A mice (g). (h-i) Representative images of the layer II/III of motor cortex show lack of co-localization of MCP1+ cells with iNOS in MCP1-CCR2-WT mice (h) and MCP1-CCR2- hSOD1G93A mice (i). Scale bar = 10 μm. (PDF 961 kb) [file 12974_2017_896_MOESM3_ESM.pdf]
