# Supplementary material for: Evidence for an early innate immune response in the motor cortex of ALS
Source: J Neuroinflammation. 2017 Jun 26;14:129. doi: 10.1186/s12974-017-0896-4 (PMC5485686; doi:10.1186/s12974-017-0896-4)
Supplement: Supplementary file 2 — MCP1+ cells and CCR2+ cells are present in the motor cortex and they are in close proximity to CSMN in the MCP1-CCR2-hSOD1G93A mice. (a) Representative image showing very few MCP1+ cells in the motor cortex of MCP1-CCR2-WT mice. (b) Representative image showing increased numbers of MCP1+ cells in the motor cortex of MCP1-CCR2-hSOD1G93A mice. MCP1+ cells express microglia marker CD11b. Insets enlarged to the right (b’-b”’). (c) Experimental design depicting retrograde transduction of CSMN approach using AAV-eGFP in the MCP1-CCR2-WT and MCP1-CCR2-hSOD1G93A mice. AAV2-eGFP was injected into the CST of mice at P30, and tissue was collected at P60. (d, e) Representative images show MCP1+ cells near transduced CSMN (eGFP+) in the layer V of the motor cortex (d, e) and in the layer II/III of the motor cortex (f, g) in MCP1-CCR2-hSOD1G93A mice. (h, j) Representative images show MCP1+ cells expressing phagocytic marker CD68 and their interaction with transduced CSMN in the layer V of motor cortex in the MCP1-CCR2-hSOD1G93A mice. (k-n) Representative image showing CCR2+ cells in layer II/III of motor cortex co-localizing with monocyte marker CD45 and infiltrating monocyte marker Ly6C. Scale bar:s: a,b,d-g =20 μm; k-n = 10 μm. (PDF 1521 kb) [file 12974_2017_896_MOESM2_ESM.pdf]

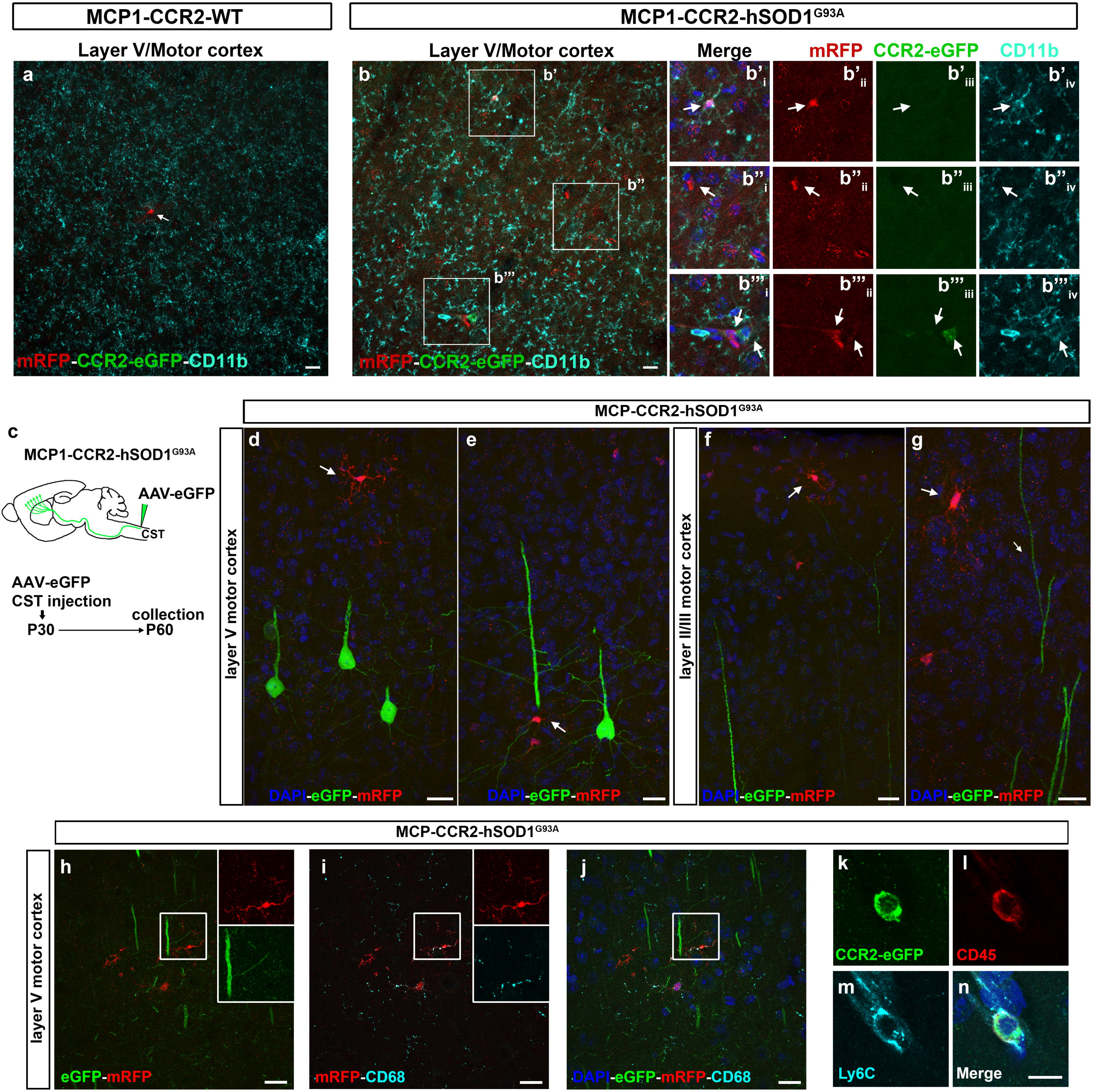

MCP1-CCR2-WT
MCP1-CCR2-hSOD1^{G93A}
Layer V/Motor cortex
Layer V/Motor cortex
Merge
mRFP
CCR2-eGFP
CD11b
a
b
b'
b''
b'''
mRFP-CCR2-eGFP-CD11b
mRFP-CCR2-eGFP-CD11b
c
MCP1-CCR2-hSOD1^{G93A}
AAV-eGFP
CST
AAV-eGFP
CST injection
P30
collection
P60
MCP-CCR2-hSOD1^{G93A}
layer V motor cortex
layer II/III motor cortex
d
e
f
g
DAPI-eGFP-mRFP
MCP-CCR2-hSOD1^{G93A}
layer V motor cortex
h
i
j
k
l
m
n
eGFP-mRFP
mRFP-CD68
DAPI-eGFP-mRFP-CD68
CCR2-eGFP
CD45
Ly6C
Merge
